# MR imaging and outcome in neonatal HIBD models are correlated with sex: the value of diffusion tensor MR imaging and diffusion kurtosis MR imaging

**DOI:** 10.3389/fnins.2023.1234049

**Published:** 2023-09-15

**Authors:** Jieaoxue Bao, Xiaoan Zhang, Xin Zhao

**Affiliations:** ^1^Department of Imaging, Third Affiliated Hospital of Zhengzhou University, Zhengzhou, China; ^2^Henan International Joint Laboratory of Neuroimaging, Zhengzhou, China

**Keywords:** diffusion kurtosis imaging, neonate, hypoxic-ischemic, neurobehavioral deficiency, sex difference

## Abstract

**Objective:**

Hypoxic-ischemic encephalopathy can lead to lifelong morbidity and premature death in full-term newborns. Here, we aimed to determine the efficacy of diffusion kurtosis (DK) [mean kurtosis (MK)] and diffusion tensor (DT) [fractional anisotropy (FA), mean diffusion (MD), axial diffusion (AD), and radial diffusion (RD)] parameters for the early diagnosis of early brain histopathological changes and the prediction of neurodegenerative events in a full-term neonatal hypoxic-ischemic brain injury (HIBD) rat model.

**Methods:**

The HIBD model was generated in postnatal day 7 Sprague-Dawley rats to assess the changes in DK and DT parameters in 10 specific brain structural regions involving the gray matter, white matter, and limbic system during acute (12 h) and subacute (3 d and 5 d) phases after hypoxic ischemia (HI), which were validated against histology. Sensory and cognitive parameters were assessed by the open field, novel object recognition, elevated plus maze, and CatWalk tests.

**Results:**

Repeated-measures ANOVA revealed that specific brain structures showed similar trends to the lesion, and the temporal pattern of MK was substantially more varied than DT parameters, particularly in the deep gray matter. The change rate of MK in the acute phase (12 h) was significantly higher than that of DT parameters. We noted a delayed pseudo-normalization for MK. Additionally, MD, AD, and RD showed more pronounced differences between males and females after HI compared to MK, which was confirmed in behavioral tests. HI females exhibited anxiolytic hyperactivity-like baseline behavior, while the memory ability of HI males was affected in the novel object recognition test. CatWalk assessments revealed chronic deficits in limb gait parameters, particularly the left front paw and right hind paw, as well as poorer performance in HI males than HI females.

**Conclusions:**

Our results suggested that DK and DT parameters were complementary in the immature brain and provided great value in assessing early tissue microstructural changes and predicting long-term neurobehavioral deficits, highlighting their ability to detect both acute and long-term changes. Thus, the various diffusion coefficient parameters estimated by the DKI model are powerful tools for early HIBD diagnosis and prognosis assessment, thus providing an experimental and theoretical basis for clinical treatment.

## 1. Introduction

Hypoxic ischemic brain damage (HIBD) is a serious neurological problem in the perinatal period. HIBD refers to neurological dysfunction of the fetal brain, which occurs due to hypoxia and ischemia before and after delivery and is an important cause of brain damage in full- and near-full-term infants. Neonatal hypoxic-ischemic encephalopathy (HIE) is an important cause of long-term neurological disability and death in newborns, occurring in 1–6 cases per 1,000 full-term live births (Shankaran, 2009). Perinatal HIE is responsible for 23% of newborn fatalities worldwide, affecting 1.5–2 per 1,000 births in wealthy nations, and up to 26 per 1,000 births in underdeveloped countries (Black et al., 2010; Kurinczuk et al., 2010; Lawn et al., 2010; Hagberg et al., 2015). HIE usually leads to long-term neurological disorders and disabilities, including cerebral palsy, behavioral problems, motor deficiency, intellectual disability, seizure, and epilepsy (Hagberg et al., 2015; Li et al., 2017; Borjini et al., 2019). Despite advances in perinatal care and the use of therapeutic hypothermia, many treated infants still suffer from lifelong attentional, social, functional motor, cognitive, and behavioral deficits (Herrera et al., 2018). To date, therapeutic hypothermia is the only neuroprotective therapy proven to improve neurodevelopmental outcomes of HIE; however, the treatment needs to be administered within the first six postnatal hours, and up to 40% of infants who have received this treatment in clinical trials have either died or suffered from long-term disabilities such as intellectual disability and cerebral palsy (Azzopardi et al., 2009). Therefore, HIE remains a major clinical problem.

The metabolic status, cerebral blood flow, and the region and timing of crucial developmental processes largely determine the susceptibility of the immature central nervous system to hypoxia-ischemia (HI) (Vexler and Ferriero, 2001). Currently, HIE is identified by biochemical and clinical measures, such as Apgar scores (Simon et al., 2023), initial lactate (Wiberg-Itzel et al., 2008; East et al., 2010), and abnormalities in basal indicators (Douglas-Escobar and Weiss, 2015), all of which have a low positive predictive value. In neonates with HIE, assessment of severity relying primarily on electroencephalogram and Sarnat grading criteria is accurate, but it needs to be performed 24 h postnatally, beyond the limited therapeutic window, making accurate and timely diagnosis difficult (Douglas-Escobar and Weiss, 2015; Awal et al., 2016; Akamatsu et al., 2019). A reliable and validated detection method for the early diagnosis, assessment, monitoring, and outcome prediction of newborns with HIE will support clinical decision-making and early targeted intervention.

HI is increasingly believed to be sexually dimorphic, in which males exhibit more persistent cognitive impairment than females (Al Mamun et al., 2018). The effects of hormones on HI are often overlooked, probably because the lesion develops in the neonatal era, well-before maturity. Specifically, high levels of testosterone relative to females may exacerbate deleterious behavioral outcomes in males following neonatal brain injury. Previous studies (Hill et al., 2011) have shown that injection of testosterone propionate in HI postnatal day (PND) seven females before HI injury enhanced the lesion volume and behavioral abnormalities, similar to the findings in HI males. Additionally, estrogen has been well-documented to be advantageous in the outcomes of adult stroke (Carwile et al., 2009; Nematipour et al., 2020), multiple sclerosis, Parkinson's disease, Alzheimer's disease (Villa et al., 2016), and experimental cerebral hemorrhage (Zheng et al., 2015). However, the ovaries are largely quiescent in neonatal P7 HI females (McCarthy, 2008). Studies suggest that sex differences in neural injury responses may originate from HI-activated cell death pathways. Indeed, neuronal and glial apoptosis in the brain is preferentially caspase-dependent and caspase-independent in females and males, respectively (Renolleau et al., 2008; Askalan et al., 2015). Knockout of the gene for poly (ADP-ribose) polymerase 1 (Parp-1), an enzyme that is present only in the caspase-independent pathway, showed significant protection against HI injury in males but not in females (Zhu et al., 2006; Charriaut-Marlangue et al., 2018; Bonnin et al., 2020, 2021). Moreover, inhibition of caspase cleavage in caspase-dependent pathways is neuroprotective in females only (Renolleau et al., 2007). Therefore, interactions with sex are crucial when assessing the outcome of prevention and treatment strategies.

Diffusion kurtosis imaging (DKI), a clinically viable extension of diffusion tensor imaging (DTI), is a novel diffusion imaging technique. Based on the quantification of non-Gaussian water diffusion, DKI more closely resembles real tissue water molecule diffusion (Jensen et al., 2005). Biological systems are primarily composed of multiple compartments, which result in heterogeneous diffusion in biological tissues due to structural hindrance and limitation; therefore, the Gaussian water diffusion probability assumed by the conventional DTI may be unsuitable for describing the actual diffusion process *in vivo*. DKI greatly enhances the sensitivity to the microstructure of biological tissues, which in turn improves the early and subtle detection of pathophysiological changes. The DKI model more accurately describes diffusion-weighted signals and mitigates the b-value dependence of diffusivity estimates compared to the DTI model (Veraart et al., 2011).

Neuroimaging is widely used to assist with the diagnosis and neurodevelopmental prediction of infants with HIE. Studies have compared DKI-related parameters with the indicators provided by DTI, including studies involving clinical research pathologies and applications, such as the developing brain (Grinberg et al., 2017; Shi et al., 2019), glioma (Raab et al., 2010; Van Cauter et al., 2012), epilepsy (Guo et al., 2022), stroke (Hui et al., 2012b; Umesh Rudrapatna et al., 2014), traumatic brain injury (Wang et al., 2018), multiple sclerosis (Raz et al., 2013), autism (McKenna et al., 2020), Alzheimer's disease (Chu et al., 2022), schizophrenia (McKenna et al., 2019), Huntington's chorea (Blockx et al., 2012), and Parkinson's disease (Bai et al., 2021). However, studies on the application of DKI to HIE are relatively rare. Here, we used a neonatal rat model of HIBD to investigate the efficacy of diffusion kurtosis (DK) parameters and diffusion tensor (DT) parameters for the early diagnosis of early brain histopathological changes and prediction of neurodegenerative events after neonatal HI to understand their potential to discriminate and characterize microstructural tissue complexity and its variability, as well as the role of sex in this process. Quantifying the pattern and degree of brain injury on neuroimaging is important to guide the application of additional neuroprotective interventions and predict neurodevelopmental outcomes.

## 2. Materials and methods

### 2.1. Animal preparations

Sprague-Dawley (SD) rats were acquired from the Laboratory Animal Center [Henan, China, License No. SCXK(Henan)2017-0001] and kept in a pathogen-free, humidity- and temperature-controlled facility with a 12-h light/dark cycle and were provided free access to food and water. The ambient temperature of the facility was 22 ± 2°C, and the constant humidity was approximately 70%. Eighty-eight healthy PND7 pups of both sexes (16–18.5 g) were selected. Animal studies were conducted according to the FELASA guidelines and 3Rs principles. All procedures followed local ethics committee rules and were endorsed and approved by the Institutional Ethics Committee for Animal Use (Application No. 2022-359-01). Every attempt was made to minimize animal suffering and the number of animals used for experiments.

### 2.2. Experimental design

The 88 pups (*n* = 44 per sex) were divided into the control group (*n* = 22 per sex) and the HI group (*n* = 22 per sex). The pups were grouped arbitrarily by sex. In each litter, we first checked the sex and attempted to assign an equal number of groups from each litter. After the corresponding model preparation in the control and HI groups, the pups were given arbitrary numbers according to their sex and assigned to different subgroups.

In Experiment 1, the continuous MRI scan group underwent continuous MRI acquisition at 12 h, 3 and 5 d after HI. Animal models in the HI group were included based on the MRI scan 3 d after HI, and the exclusion criteria were mild or severe brain injury and death during MRI acquisition. The experiment involved the control group (*n* = 7 per sex) and the HI group (*n* = 7 per sex).

In Experiment 2, the exclusion criteria for pups in the behavioral group were the same as above. Pups were divided into cages at PND 23 and behavioral tests were performed from PND 60 onward. The experiment involved the control group (*n* = 6 per sex) and the HI group (*n* = 6 per sex).

In Experiment 3, pups in the stained group were excluded based on MRI scans at 12 h and 3 d after HI, with the same exclusion criteria as above. Males and females that met the inclusion criteria for brain tissue specimen collection were randomly selected at each time point from the control and HI groups. The tissues were subjected to H&E and TUNEL staining at three-time points, each involving the control group (*n* = 3 per sex) and HI group (*n* = 3 per sex).

### 2.3. Neonatal HI injury model

PND7 pups were randomly assigned to undergo either sham or HI surgery. HI animals were anesthetized (4–5% isoflurane induction, 1.5–2% maintenance) and had their right common carotid artery exposed and permanently double-ligated with 5/0 thread. The entire surgical procedure was completed within 5 min. Pups were placed on a heating pad (37°C) for 10 min to recover from anesthesia before returning to the dam. Two hours after surgery, pups were placed in a hypoxic chamber at 37°C with an oxygen fraction (FIO_2_: 0.08) for 90 min. The mortality rate of HI rats was < 5%. Sham-operated animals, kept under normal conditions (FIO_2_: 0.21), had their right carotid artery isolated after anesthesia without arterial ligation. At the end of the hypoxic period, all pups were returned to their dams.

### 2.4. Magnetic resonance imaging acquisition

*In vivo* imaging experiments were conducted on a horizontal 4.7 T cryogen-free (dry magnet) preclinical MR system (MR Solutions, Guildford, Surrey, United Kingdom) using a quadrature rat brain coil for both excitation and signal detection. During imaging, rats were anesthetized using isoflurane (4% induction, 1.5% maintenance) (RWD Life Science Co., Ltd.).

Anatomical images were acquired using an axial T2-weighted Fast Spin Echo images: 20 slices, slice thickness = 1.0 mm, no slice gap, matrix = 256 × 238, FOV = 25 × 25 mm^2^, spatial resolution: 98 × 105 μm^2^, TR = 4,000 ms, effective TE = 51 ms, echo train = 7, average = 3, and scan time = 7 min.

The DKI protocol included the acquisition of 3 b-values (0, 1,000, 2,000 s/mm^2^) and 12 non-collinear diffusion gradient directions. Images were collected with a two-shot SE-EPI sequence with one navigator and readout interleave mode (20 slices, slice thickness = 1 mm, no gap, TR/TE = 4,000/23 ms, δ = 4 ms, Δ = 11 ms, acquisition matrix = 60 × 40, zero filled to 64 × 64, spatial resolution: 313 × 313 μm^2^, NEX = 4, receiver bandwidth = 200 kHz, total scan time = 38 min 40 s).

DTI images were acquired with a 2-shot spin-echo EPI sequence with one navigator and read interleave mode: 1 b0 and 66 directions of b = 1,000 s/mm^2^, TR = 5,000 ms, TE = 27 ms, δ = 5 ms, Δ = 13 ms, NEX = 1, matrix = 100 × 70 (partial Fourier along Phase encoding), reconstructed matrix = 128 × 128, slice thickness = 1 mm, number of slices = 20, receiver bandwidth = 200 kHz, in-plane spatial resolution (0.172 × 0.172) mm^2^, and total scan time = 22 min, 40 s.

### 2.5. Region of interest selection and image processing

**Figure 1:**
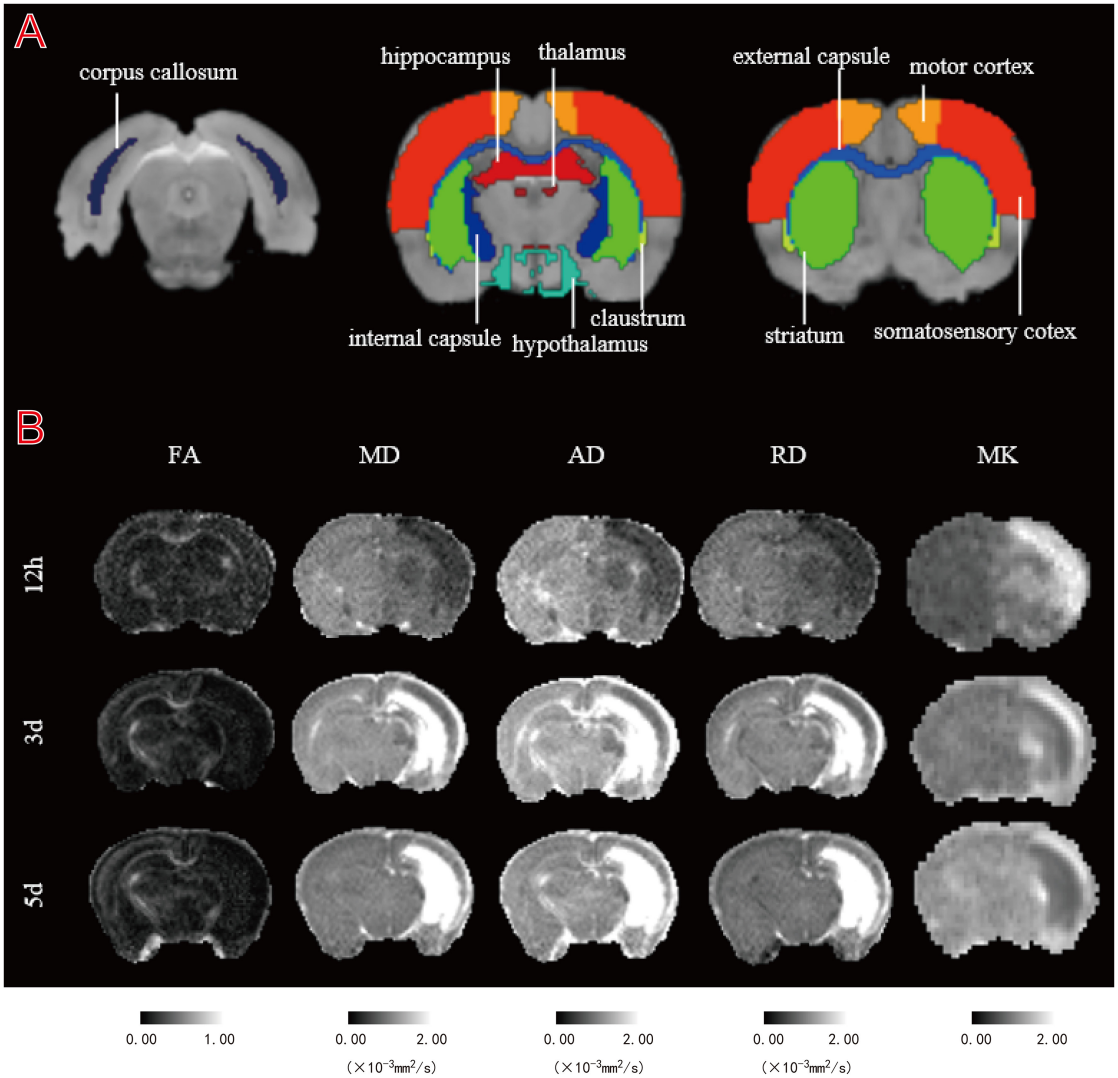
Illustration of regions of interest (ROIs) delineation and representative DT and DK parametric maps. **(A)** Representative images of ROIs. The regions shown are the bilateral corpus callosum (cc), internal capsule (ic), external capsule (ec), hypothalamus (hyp), striatum (str), claustrum (cla), thalamus (tha), motor cortex (mc), somatosensory cortex (sc), and hippocampus (hip). **(B)** Fractional anisotropy (FA), mean diffusion (MD), axial diffusion (AD), radial diffusion (RD), and mean kurtosis (MK) maps of a representative HI rat at 12 h, 3 and 5 d.

The eddy current correction and EPI correction are completed by TORTOISE software. After preprocessing, a symmetric image normalization method (SyN) in ANTs was used to non-linearly register the brain MRI atlases of 14-day-old rats to the DTI and DKI image data of each rat brain (b = 0 s/mm^2^). ROI, including the corpus callosum (cc), internal capsule (ic), external capsule (ec), hypothalamus (hyp), striatum (str), claustrum (cla), thalamus (tha), motor cortex (mc), somatosensory cortex (sc), and hippocampus (hip), were then extracted from the brain atlas (Figure 1A). Parameter maps of fractional anisotropy (FA), mean diffusion (MD), axial diffusion (AD), and radial diffusion (RD) based on DTI images were calculated by MRtrix3 (https://www.mrtrix.org/). Mean kurtosis (MK) values based on DKI images were calculated using the DIPY tool programmed in Python (version 3.8.5). The parameter maps obtained from MRtrix3 and DIPY calculations were aligned to the brain atlases of the individual subjects to obtain the corresponding parameter values. An uninformed researcher delineated the lesions based on MD and MK parameter maps by means of ITK-SNAP software. The mirror regions of the lesions in the contralateral brain were determined using standard brain symmetry mapping. We obtained the mean values of the parameters for the lesions and each brain ROI for subsequent statistical calculations. We also calculated the percentage change from normal to HI tissue for different parameters using [(L–C)/C] × 100%, where L and C are the mean values of ROIs in the HI and contralateral hemispheres, respectively.

### 2.6. Behavioral tests

Behavioral tests were applied from PND 61 onward. All of the behavioral tests were conducted in an enclosed, quiet, dimly lit laboratory with constant light, temperature, and humidity. All of the behavioral tests except gait analysis were conducted using ANY-maze 7.20 (Stoelting Co.) for animal tracking and data analysis. After each test, the experimental equipment was cleaned with 75% alcohol to decrease the impact of odor on the subsequent test.

#### 2.6.1. Open field test (OFT; PND 61)

The OFT is commonly used to assess an animal's autonomous, exploratory, and anxious behavior in unfamiliar environments. The open field apparatus used in this experiment was a black plastic box (100 × 100 × 40 cm), the bottom of which was equally divided into 16 small squares, with four central squares labeled “central” and the rest labeled “peripheral.” The rats were placed on one side of the field, and the movement of each animal was tracked for 10 min using a camera above the center of the arena. Different characteristics of the locomotor activity were recorded, including distance traveled, average speed, resting time, and time spent in the central and peripheral area.

#### 2.6.2. Novel object recognition test (NOR; PND 62)

Rats were assessed for recognition memory using the NOR test. This task was performed under the same environmental conditions as the OFT. The task was divided into three sections: familiarization, training, and testing, with a 1-h interval between training and testing. In the training phase, the researchers placed two identical objects near the corner of one wall in the arena. In the test phase, one of the objects was replaced with a new object. Each test rat was placed on one side of the arena and allowed to explore the object for 5 min. The time spent exploring each object was recorded, and the object preference ratio (the ratio of time spent exploring one object to the total time spent exploring both objects) and recognition index (the ratio of the difference between the time spent exploring two objects to the total time spent exploring both objects) were calculated.

#### 2.6.3. Elevated plus-maze test (EPM; PND 63)

The apparatus was a plus “+”-shaped platform with a central area (10 × 10 cm), two closed arms (50 × 5 cm), and two open arms (50 × 5 cm, no walls) surrounded by a 15-cm-high wall. The maze was raised 50 cm above the ground. Initially, the rats were placed in the open arm area near the center with their heads facing the closed arm and were allowed to explore freely for 5 min in a quiet environment. The time and number of entries in the closed and open arms were recorded, and the anxiety index was calculated as the ratio of the difference in time between the closed and open arms to their total time.

#### 2.6.4. Gait analysis (PND 71)

Gait and postural parameters were analyzed at PND71 using the CatWalk XT system (Noldus Information Technology, Wageningen, the Netherlands). The CatWalk system was comprised of a 1.3-meter-long horizontal glass panel covered with a detachable tunnel, which casted a weak light on the walkway. Rats were placed at the start of the pathway and willingly traversed the plate to their cage. As their paws touch the glass plate, the light is refracted to the opposite side. The movement is then captured by a high-speed camera below the platform and transmitted to the CatWalk XT software for parametric analysis. After 5 consecutive days of training (PND 66-70), the rats must perform at least three uninterrupted runs to be eligible for CatWalk analysis on test day at PND 71.

### 2.7. Histology

In the acute (12 h) and subacute (3 and 5 d) phases after HI, randomly selected pups were transcardially perfused with 4% paraformaldehyde before removing the brains and placing them in 4% formaldehyde for more than 24 h. Corresponding to the T2WI scanning position, tissues containing the anatomical layer to be observed were cut for paraffin embedding, and 4-μm coronal brain sections were prepared with a paraffin slicer. Brain tissue sections were stained with terminal deoxynucleotidyl transferase-mediated notch end labeling (TUNEL) and hematoxylin-eosin (H&E).

### 2.8. Statistical analysis

All of the statistical analyses were performed using SPSS 26.0 software (SPSS, Chicago, IL, USA). Our sample size was based on that outlined in previous reports rather than using statistical methods to predetermine the sample size (Borjini et al., 2019; He et al., 2020). All of the datasets were first tested for normality before choosing the statistical tests. For normally distributed data, statistical comparisons between two groups were performed using either an unpaired Student's *t*-test or a paired *t*-test. Multiple comparisons among more than two groups were made using the two-way repeated measure ANOVA (rm ANOVA) or two-way ANOVA followed by the Bonferroni *post-hoc* test. For non-normally distributed data, statistical comparisons between two groups were performed using the Mann-Whitney *U*-test or the Wilcoxon paired signed-rank test. Multiple comparisons among more than two groups were made using the Kruskal-Wallis test followed by the Dunn's test. The data are presented as the mean ± SEM, and significance was assumed when *p* < 0.05.

## 3. Results

### 3.1. Imaging manifestations of brain tissue

Most HI group lesions were located in the cortex and subcortical white matter regions. All animals survived the 5-day test following HI insult. Figure 1B shows the FA, MD, AD, RD, and MK maps from a representative rat at three time points after HI injury. Compared to controls, the MD, AD, and RD maps in the lesion area showed relatively uniform low signal during the acute stage (12 h), while the FA maps showed no significant sign. In the subacute stage (3 and 5 d), the FA maps showed a relatively uniform low signal, while the MD, AD, and RD maps showed an uneven high signal, with a gradually expanding area of high signal. Moreover, 12 h after HI, we observed a relatively uniform high signal in and around the injury site on the MK map, followed by a gradual decrease in the area of high signal over a 5-day period, showing a heterogeneous high signal. Notably, the regions of inhomogeneous high signal exhibited by the MD, AD, RD, and MK maps effectively complemented each other, as shown by their respective maps.

### 3.2. DK-DT analysis

Statistical values are summarized in Table 1 for the regional analysis of HI insult, Supplementary Table 1 for the cortical gray matter, white matter, hippocampal, and deep gray matter analyses. The time effects, HI effects, sex effects, and interactions (HI^*^sex, time^*^HI, time^*^sex, time^*^HI^*^sex) are shown.

**Table 1:** Summary of the statistical results from repeated measurement ANOVA (rm ANOVA) of DT/DK parameters in the lesion region after HI injury.

**ROI**	**ANOVA**	**DK-parameters**	**DT-parameters**			
		**MK**	**FA**	**MD**	**AD**	**RD**
Lesion	Time effect	*F*_2, 23_ = 79.094, ***p*** **≤0.0001**	*F*_2, 23_ = 6.220, ***p*** **=** **0.007**	*F*_2, 48_ = 13.625, ***p*** **≤0.0001**	*F*_2, 48_ = 11.855, ***p*** **≤0.0001**	*F*_2, 48_ = 14.137, ***p*** **≤0.0001**
	Time^*^HI	*F*_2, 23_ = 56.670, ***p*** **≤0.0001**	*F*_2, 23_ = 3.460, ***p*** **=** **0.049**	*F*_2, 48_ = 41.268, ***p*** **≤0.0001**	*F*_2, 48_ = 42.099, ***p*** **≤0.0001**	*F*_2, 48_ = 39.361, ***p*** **≤0.0001**
	Time^*^sex	*F*_2, 23_ = 2.335, *p* = 0.119	*F*_2, 23_ = 7.058, ***p*** **=** **0.004**	*F*_2, 48_ = 1.527, *p* = 0.227	*F*_2, 48_ = 1.051, *p* = 0.357	*F*_2, 48_ = 1.837, *p* = 0.17
	Time^*^HI^*^sex	*F*_2, 23_ = 2.174, *p* = 0.137	*F*_2, 23_ = 0.217, *p* = 0.807	*F*_2, 48_ = 2.353, *p* = 0.106	*F*_2, 48_ = 2.084, *p* = 0.136	*F*_2, 48_ = 2.425, *p* = 0.099
	HI effect	*F*_1, 24_ = 187.626, ***p*** **≤0.0001**	*F*_1, 24_ = 11.714, ***p*** **=** **0.002**	*F*_1, 24_ = 7.07, ***p*** **=** **0.014**	*F*_1, 24_ = 17.191, ***p*** **≤0.0001**	*F*_1, 24_ = 2.492, *p* = 0.128
	Sex effect	*F*_1, 24_ = 2.827, *p* = 0.106	*F*_1, 24_ = 4.461, ***p*** **=** **0.045**	*F*_1, 24_ = 0.736, *p* = 0.399	*F*_1, 24_ = 1.822, *p* = 0.19	*F*_1, 24_ = 0.266, *p* = 0.611
	HI^*^sex	*F*_1, 24_ = 0.001, *p* = 0.973	*F*_1, 24_ = 0.002, *p* = 0.968	*F*_1, 24_ = 1.209, *p* = 0.282	*F*_1, 24_ = 1.015, *p* = 0.324	*F*_1, 24_ = 1.211, *p* = 0.282

*P* < 0.05 was considered statistically significant. Bold values represent significant *p*-values.

#### 3.2.1. Lesions

DT parameters (FA, MD, AD, and RD) and DK parameters (MK) in the lesion almost always showed significant time effects, HI effects, and time^*^HI interactions, as well as non-significant sex effects, HI^*^sex interactions, time^*^sex interactions, and time^*^HI^*^sex interactions (Table 1). Notably, the time^*^HI interaction became the main effect affecting DT and DK parameters, while the time^*^sex interaction was another major effect affecting FA.

#### 3.2.2. Brain structures

##### 3.2.2.1. DT-parameters

For FA, we found no significant HI^*^sex interactions, time^*^HI interactions, or time^*^HI^*^sex interactions across all of the measured brain structures (Supplementary Table 1). Notably, the time^*^sex interaction was the main effect affecting all of the measured structures in the white matter and deep gray matter, except that no effect was found to have a significant effect on the hyp. Additionally, the HI effect and the time^*^sex interaction together had a major effect on the hip. Moreover, the time effect, as a main effect, mainly affected the mc, while the separate effects of time and sex became the main effects affecting the sc.

For MD, AD, and RD, we found no significant sex effects in all of the measured brain structures. Notably, the time^*^HI interactions for MD, AD, and RD were all significant at the cortical, white matter, and hippocampal levels (Supplementary Table 1), whereas performance varied in deep gray matter, with interactions being significant for MD and AD in the str and cla, and for AD in the tha. We also observed that the time^*^HI^*^sex interaction had significant effects on the MD, AD, and RD in the sc and AD in the hyp, while the time^*^sex interaction only had a significant effect on RD in the hip. Simultaneously, no significant effect was found on RD at the deep gray matter level, with similar performance for MD in the hyp and tha.

##### 3.2.2.2. DK-parameters

MK showed significant time effects, HI effects, and time^*^HI interactions in almost all of the measured brain structures (Supplementary Table 1). The time^*^HI interaction acted as a main effect, affecting the rest of the brain structures except the mc and hyp. Simultaneously, we noticed that the time^*^HI^*^sex interaction was another major effect affecting the str, while the HI^*^sex interaction was another main effect affecting the ic, str, and cla. However, the main effects affecting the mc and hyp were separate effects of time, HI, and sex.

### 3.3. Temporal evolution of DK and DT parameters

#### 3.3.1. Lesions

##### 3.3.1.1. DT-parameters

**Figure 2:**
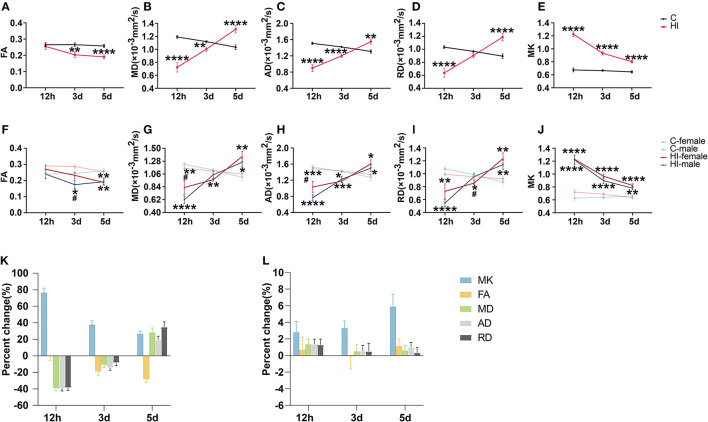
Assessment of diffusion tensor (DT) and diffusion kurtosis (DK) metrics in the focal area after HI injury. **(A–E)** Effect of time and HI on DT and DK metrics. **(F–J)** Effects of time, HI and sex on DT and DK metrics. Graphs show FA **(A, F)**, MD **(B, G)**, AD **(C, H)**, RD **(D, I)**, and MK **(E, J)** of the focal area. **(K, L)** Percentage changes of different diffusion metrics at three-time points. Graphs show the focal area in the HI group **(K)** and the corresponding area in the control group **(L)**, respectively. **(A–L)** Data are presented as means ± SEM (two-way rm ANOVA or two-way ANOVA followed by the Bonferroni *post-hoc* test). *P < 0.05, **P < 0.01, ***P < 0.001, ****P < 0.0001; ^#^*P* < 0.05. *HI group vs. control group, HI-female vs. C-female, HI-male vs. C-male; ^#^HI-female vs. HI-male.

FA, MD, AD, and RD exhibited significant time^*^HI interactions in the lesion. Compared to controls, HI pups showed significantly lower MD, AD, and RD (all *p* < 0.0001) values in the acute phase (12 h) (Figures 2B–D), before increasing gradually with time and remaining lower in the subacute phase (3 d), showing significant MD (*p* < 0.01) and AD (*p* < 0.0001) only (Figures 2B, C), while the MD (*p* < 0.0001), AD (*p* < 0.01), and RD (*p* < 0.0001) values were significantly higher in the subacute phase (5 d) (Figures 2B–D). The FA of HI pups was not significantly different from that of controls in the acute phase (12 h), decreased gradually with time, and was significantly lower than that of controls in the subacute phase (3 and 5 d) (*p* < 0.01; *p* < 0.0001) (Figure 2A).

##### 3.3.1.2. DK-parameters

MK exhibited significant time^*^HI interactions in the lesion. Compared to controls, the MK (*p* < 0.0001) values in HI pups were significantly higher in the acute phase (12 h), decreased gradually with time, and remained significantly higher (*p* < 0.0001) in the subacute phase (3 and 5 d) (Figure 2E).

#### 3.3.2. Brain structures

**Figure 3:**
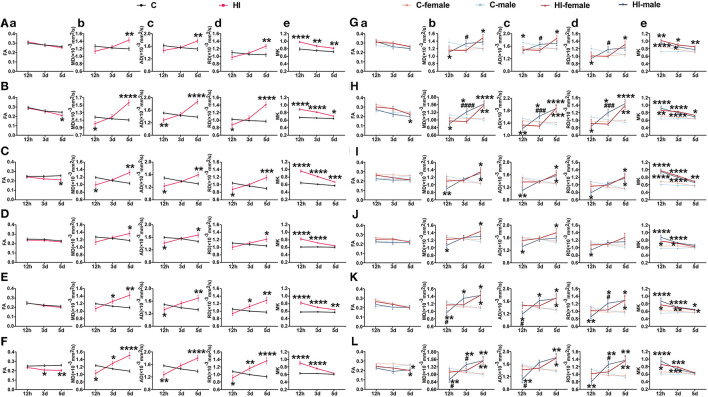
Assessment of DT and DK metrics at the level of cortical gray matter, white matter and hippocampus after HI injury. **(A–F)** Effect of time and HI on DT and DK metrics. **(G–L)** Effects of time, HI and sex on DT and DK metrics. Graphs show FA (a), MD (b), AD (c), RD (d), and MK (e) at the levels of mc **(A, G)**, sc **(B, H)**, cc **(C, I)**, ic **(D, J)**, ec **(E, K)**, and hip **(F, L)**, respectively. **(A–L)** Data are presented as means ± SEM (two-way rm ANOVA or two-way ANOVA followed by the Bonferroni *post-hoc* test). *P < 0.05, **P < 0.01, ***P < 0.001, ****P < 0.0001; ^#^*P* < 0.05, ^###^*P* < 0.001, ^####^*P* < 0.0001. *HI group vs. control group, HI-female vs. C-female, HI-male vs. C-male; ^#^HI-female vs. HI-male.

Changes in DK and DT parameters were quantified simultaneously at different time points for each brain region in the acute (12 h) and subacute (3 and 5 d) phases following HI injury (Figures 3A–F; Supplementary Figures 1A–D). For MK, all of the brain structures, except the mc and hyp, showed significant time^*^HI interaction. For MD, AD, and RD, the time^*^HI interaction was significant in the cortical, white matter, and hippocampal levels, except for the deep gray matter, where it varied. During the acute phase (12 h), we observed significant changes (*p* < 0.001, *p* < 0.0001) in MK values in all of the selected brain structures (Figures 3A–F; Supplementary Figures 1A–D), while the MD, AD, and RD values showed significant changes (*p* < 0.05, *p* < 0.01) at the level of the sc, white matter, and hip (Figures 3B–F), with trends consistent with the lesion. Notably, only the FA values in the cc and hip showed similar trends to the lesion after HI injury, with significant changes at 3 and 5 d during the subacute phase, respectively (*p* < 0.05; *p* < 0.05, *p* < 0.01) (Figures 3C, F). No significant time^*^HI interactions were found for FA in previous analyses of DT-DK parameters of brain regions, so we will not describe FA here.

Additionally, for MD, AD, and RD, we found a significant time^*^HI^*^sex interaction in the sc. Compared to control males, the MD (*p* < 0.05), AD (*p* < 0.01), and RD (*p* < 0.05) values of HI males were significantly lower in the acute phase (12 h), and progressively increased in the subacute phase (3 and 5 d), which were significantly higher than those of the controls (*p* < 0.05; *p* < 0.0001) (Figure 3H). Compared to control females, the MD (*p* < 0.01), AD (*p* < 0.001), and RD (*p* < 0.01) values were significantly higher in HI females only during the subacute phase (5 d) (Figure 3H). Notably, compared to HI females, the MD (*p* < 0.0001), AD (*p* < 0.001), and RD (*p* < 0.001) values were significantly higher in HI males during the subacute phase (3 d), with no significant difference in the subacute phase (5 d) (Figure 3H). Similar observations were made for AD in the hyp, and for MK in the str.

### 3.4. Effect of sex on DK and DT parameters after HI injury

To further determine the effect of sex and HI on the derived parameters, a two-way ANOVA was performed on the parameters at three time points, showing differences between groups (HI females vs. control females, HI males vs. control males, and HI females vs. HI males) (Table 2; Supplementary Table 2).

**Table 2:** Summary of the statistical results from two-way ANOVA of DT/DK parameters in the lesion region at three time points after HI injury.

**ROI**	**ANOVA**	**DK-parameters**	**DT-parameters**			
		**MK**	**FA**	**MD**	**AD**	**RD**
12 h	HI effect	*F*_1, 24_ = 135.616, ***p*** **≤0.0001**	*F*_1, 24_ = 0.237, *p* = 0.631	*F*_1, 24_ = 42.452, ***p*** **≤0.0001**	*F*_1, 24_ = 52.14, ***p*** **≤0.0001**	*F*_1, 24_ = 35.815, ***p*** **≤0.0001**
	Sex effect	*F*_1, 24_ = 1.23, *p* = 0.278	*F*_1, 24_ = 3.696, *p* = 0.067	*F*_1, 24_ = 0.955, *p* = 0.338	*F*_1, 24_ = 1.841, *p* = 0.187	*F*_1, 24_ = 0.538, *p* = 0.47
	HI^*^sex	*F*_1, 24_ = 0.753, *p* = 0.394	*F*_1, 24_ = 0.234, *p* = 0.633	*F*_1, 24_ = 3.817, *p* = 0.062	*F*_1, 24_ = 3.275, *p* = 0.083	*F*_1, 24_ = 4.034, *p* = 0.056
3 d	HI effect	*F*_1, 24_ = 75.296, ***p*** **≤0.0001**	*F*_1, 24_ = 10.885, ***p*** **=** **0.003**	*F*_1, 24_ = 10.354, ***p*** **=** **0.004**	*F*_1, 24_ = 19.576, ***p*** **≤0.0001**	*F*_1, 24_ = 3.923, *p* = 0.059
	Sex effect	*F*_1, 24_ = 3.775, *p* = 0.064	*F*_1, 24_ = 6.968, ***p*** **=** **0.014**	*F*_1, 24_ = 1.889, *p* = 0.182	*F*_1, 24_ = 0.075, *p* = 0.787	*F*_1, 24_ = 4.076, *p* = 0.055
	HI^*^sex	*F*_1, 24_ = 0.093, *p* = 0.763	*F*_1, 24_ = 0.199, *p* = 0.66	*F*_1, 24_ = 0.855, *p* = 0.364	*F*_1, 24_ = 0.425, *p* = 0.521	*F*_1, 24_ = 1.089, *p* = 0.307
5 d	HI effect	*F*_1, 24_ = 37.315, ***p*** **≤0.0001**	*F*_1, 24_ = 17.609, ***p*** **≤0.0001**	*F*_1, 24_ = 16.216, ***p*** **≤0.0001**	*F*_1, 24_ = 10.524, ***p*** **=** **0.003**	*F*_1, 24_ = 19.129, ***p*** **≤0.0001**
	Sex effect	*F*_1, 24_ = 0.08, *p* = 0.78	*F*_1, 24_ = 0.081, *p* = 0.778	*F*_1, 24_ = 1.307, *p* = 0.264	*F*_1, 24_ = 1.364, *p* = 0.254	*F*_1, 24_ = 1.206, *p* = 0.283
	HI^*^sex	*F*_1, 24_ = 1.758, *p* = 0.197	*F*_1, 24_ = 0, *p* = 0.985	*F*_1, 24_ = 0.086, *p* = 0.772	*F*_1, 24_ = 0.074, *p* = 0.787	*F*_1, 24_ = 0.089, *p* = 0.768

*P* < 0.05 was considered statistically significant. Bold values represent significant *p*-values.

#### 3.4.1. Lesions

For DT parameters (FA, MD, AD, and RD) and DK parameters (MK), we found a significant HI effect in lesions at almost all three time points, as well as a non-significant sex effect and a HI^*^sex interaction (Table 2). With the exception of the acute (12 h) and subacute (3 d) phases, we found no significant effects on FA and RD, respectively. Notably, we observed a significant sex effect for FA only during the subacute phase (3 d). The FA values exhibited significantly smaller values for HI males than HI females (*p* < 0.05) (Figure 2F). In the acute phase (12 h), MD and AD values were significantly greater (*p* < 0.05) for HI females than HI males (Figures 2G, H), while in the subacute phase (3 d), RD values were significantly greater (*p* < 0.05) for HI males than HI females (Figure 2I), but with no significant sex effect. In addition, MK values show no significant differences between males and females (Figure 2J).

#### 3.4.2. Brain structures

##### 3.4.2.1. DT-parameters

Effects of sex and HI on derived parameters were quantified separately at different time points for each brain region in the acute (12 h) and subacute (3 and 5 d) phases following HI injury (Figures 3G–L; Supplementary Figures 1E–H). For FA, sex effects showed significant performance in the acute (12 h) and subacute (3 d) phases, while HI effects showed significant performance in the subacute (5 d) phase (Supplementary Table 2). In the subacute phase (3 d), the sex effect had a major impact on the cla, with significantly lower FA values in HI males compared to HI females (Supplementary Figure 1G). Although significant sex effects were found in other brain structures during the acute (12 h) and subacute (3 d) phases, there were no significant differences between HI males and females.

For MD, AD, and RD, the HI effect and the HI^*^sex interaction were significant at all three time points, whereas the sex effect and the separate effects of HI and sex were significant as main effects only during the subacute (3 d) phase (Supplementary Table 2). In the subacute phase (3 d), the sex effect had a major impact on the MD, AD, and RD values in the mc and hyp, with the exception of RD values in the hyp, which were significantly greater (*p* < 0.05) in HI males than HI females (Figure 3G; Supplementary Figure 1E). The separate effects of HI and sex mainly affected MD and RD values in the hip during this period, showing significantly greater values (*p* < 0.01; *p* < 0.05) in HI males than controls and HI females (Figure 3L). Additionally, in the acute phase (12 h), we observed significant HI^*^sex interactions for MD values in the ec and tha, as well as MD, AD, and RD values in the str, showing significantly smaller values in HI males than were observed in controls and HI females (*p* < 0.05, *p* < 0.001; *p* < 0.05) (Figure 3K; Supplementary Figures 1F, H). Meanwhile, in the subacute phase (3 d), the performance of MD, AD, and RD in the sc was similar, showing significantly larger values in HI males than controls and HI females (*p* < 0.05; *p* < 0.001, *p* < 0.0001) (Figure 3H). Although we observed a significant HI^*^sex interaction for RD values in the ec during the acute phase (12 h), no significant difference was observed on pairwise tests (Figure 3K), with similar performance in the hyp during the acute (12 h) and subacute (5 d) phases (Supplementary Figure 1E).

##### 3.4.2.2. DK-parameters

For MK, the HI effect was significant at all three time points, especially in the acute (12 h) and subacute (3 d) phases for all of the measured brain structures. In contrast, the separate effects of HI and sex, and the HI^*^sex interaction, as the main effects, were significant only in the acute (12 h) and subacute (3 d) phases (Supplementary Table 2). Although significant separate effects of HI and sex were found in the cc, hyp, mc, and hip, and significant HI^*^sex interactions were found in the ic, str, cla, and tha, the differences between HI males and females were not significant.

### 3.5. Percentage change in DK and DT parameters

Figures 2K, L show the percentage change in DK and DT parameters for the damaged area in the HI group and the corresponding area in the control group, respectively. The percentage change in MK was significantly higher than that of DT parameters during the acute phase (12 h) (Figure 2K). MK showed the largest percentage change during the acute phase (12 h). Furthermore, the change rate of MK continued to decrease positively from the acute phase (12 h) to the subacute phase (5 d), while the change rate of FA gradually increased negatively. Meanwhile, the change rates of MD, AD, and RD gradually changed from negative to positive values. By the subacute phase (5 d), the change rate of DT parameters was progressively higher than that of MK. Figure 2L shows that the changes in MK and DT parameters in the corresponding areas of controls were not significant at the three time points.

All of the brain structures except the hyp showed similar trends of changes to the lesion (Supplementary Figures 2B–J). Although similar changes were observed in the mc, MK did not change as sharply as DT parameters during the acute phase (12 h) (Supplementary Figure 2A). In the acute phase (12 h), the degree of MK change was as follows, in descending order: cc > hip > ic > ec > str > sc > tha > cla > hyp > mc.

### 3.6. Long-term neurofunctional outcomes following HI injury

To further investigate the long-term neurological damage effects of neonatal HI, we conducted complex behavioral studies in 2-month-old rats, including spontaneous movements in the open field, novel object recognition, elevated plus-maze, and CatWalk system tests to assess sensory and cognitive function. Both sex and HI injury conditions affected the various motor outcomes for the behavioral tests described above. The effects of both factors on each motor-related variable in the OFT, NOR, and EPM tests are described in Supplementary Table 3, respectively. Those behaviors significantly influenced by sex and HI injury conditions are shown in the graphs to facilitate visual comprehension.

#### 3.6.1. Anxiolytic hyperactivity-like baseline behavior in females after HI insult

**Figure 4:**
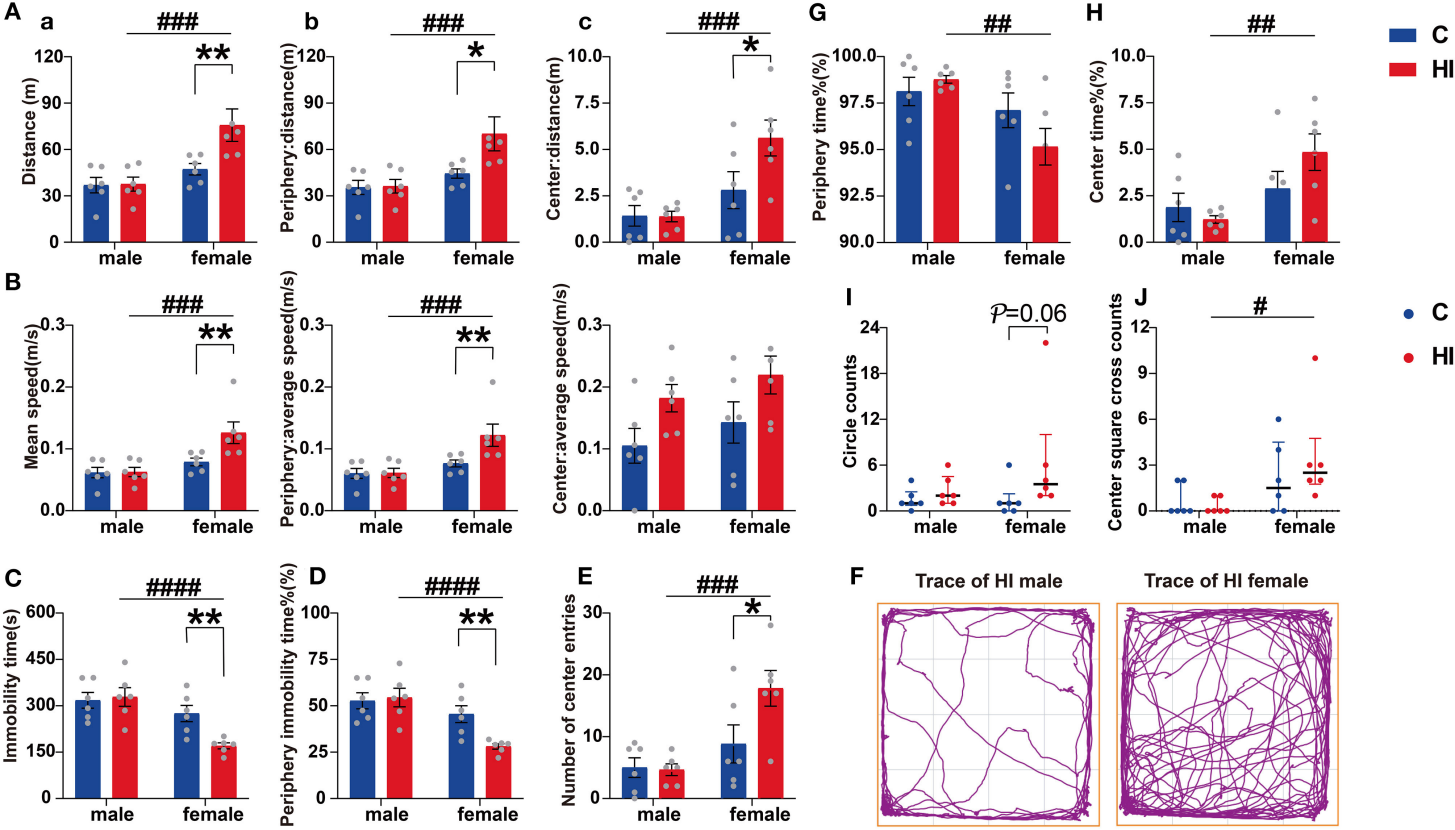
Evaluation of open-field performance in PND61 rats. **(A)** Distance of activity. **(B)** Mean speed. Graphs show the overall (a), peripheral (b), and central (c) regions, respectively. **(C)** Immobility time. **(D)** Percentage of peripheral immobility time. **(E)** Number of center entries. **(F)** Representative traces of HI male and female rat movement during the open-field test. **(G)** Percentage of periphery time. **(H)** Percentage of center time. **(I)** Circle counts. **(J)** Center square cross counts. **(A–H)** Data are presented as means ± SEM (two-way ANOVA followed by the Bonferroni *post-hoc* test). **(I, J)** Data are presented as median ± IQR (Kruskal–Wallis test followed by the Dunn's test). *P < 0.05, **P < 0.01; ^#^*P* < 0.05, ^##^*P* < 0.01, ^###^*P* < 0.001, ^####^*P* < 0.0001. *HI-female vs. C-female, HI-male vs. C-male; ^#^HI-female vs. HI-male (*n* = 6 per group).

Concerning the OFT, those behaviors significantly affected by any of our factors are shown in Figure 4. In brief, HI females moved faster, covered longer distances, and spent less time at rest during the adaptation phase of the OFT compared to controls (all *p* < 0.01) (Figures 4A–C). HI females also showed this pattern in terms of speed (*p* < 0.001), total distance (*p* < 0.001), and resting state (*p* < 0.0001) compared to HI males (Figures 4A–C). Further, we found a main effect of the sex^*^HI interaction on this pattern (Supplementary Table 3; *p* < 0.05), and we found that this pattern of movement was more pronounced in the region near the wall (Figures 4A, B, D), with a significant sex effect (Supplementary Table 3; *p* < 0.01).

Although HI females showed a significant increase in activity distance in both unprotected central and peripheral areas compared to HI males, HI female rats spent more time exploring unprotected central areas and less time in contact with the wall (both *p* < 0.01) than males (Figures 4G, H), showing anxiolytic-like baseline behaviors. This difference was also seen in the number of entries to the central area (*p* < 0.001) (Figure 4E). Additionally, the sex effects were significant (Supplementary Table 3; *p* = 0.001). To explain the differences arising from this phenomenon, we further explored the trajectory preferences of animals in the OFT. Compared to HI males, HI females showed comparable numbers in peripheral cycles (Figure 4I; *p* = 0.06) and central square crossings (Figure 4J; *p* < 0.05).

Regarding anxiety, we also observed that HI females had significantly increased entries (Supplementary Figure 3D; *p* < 0.05) in the open arm, with a significant HI effect and suggestion of a sex effect (Supplementary Table 3; *p* < 0.05, *p* = 0.07), as assessed by the EPM test. In the motor and anxiety-related behavioral measures, including the OFT and the EPM, no differences were observed between HI males and controls.

#### 3.6.2. Sex differences in recognition memory capacity after HI injury

**Figure 5:**
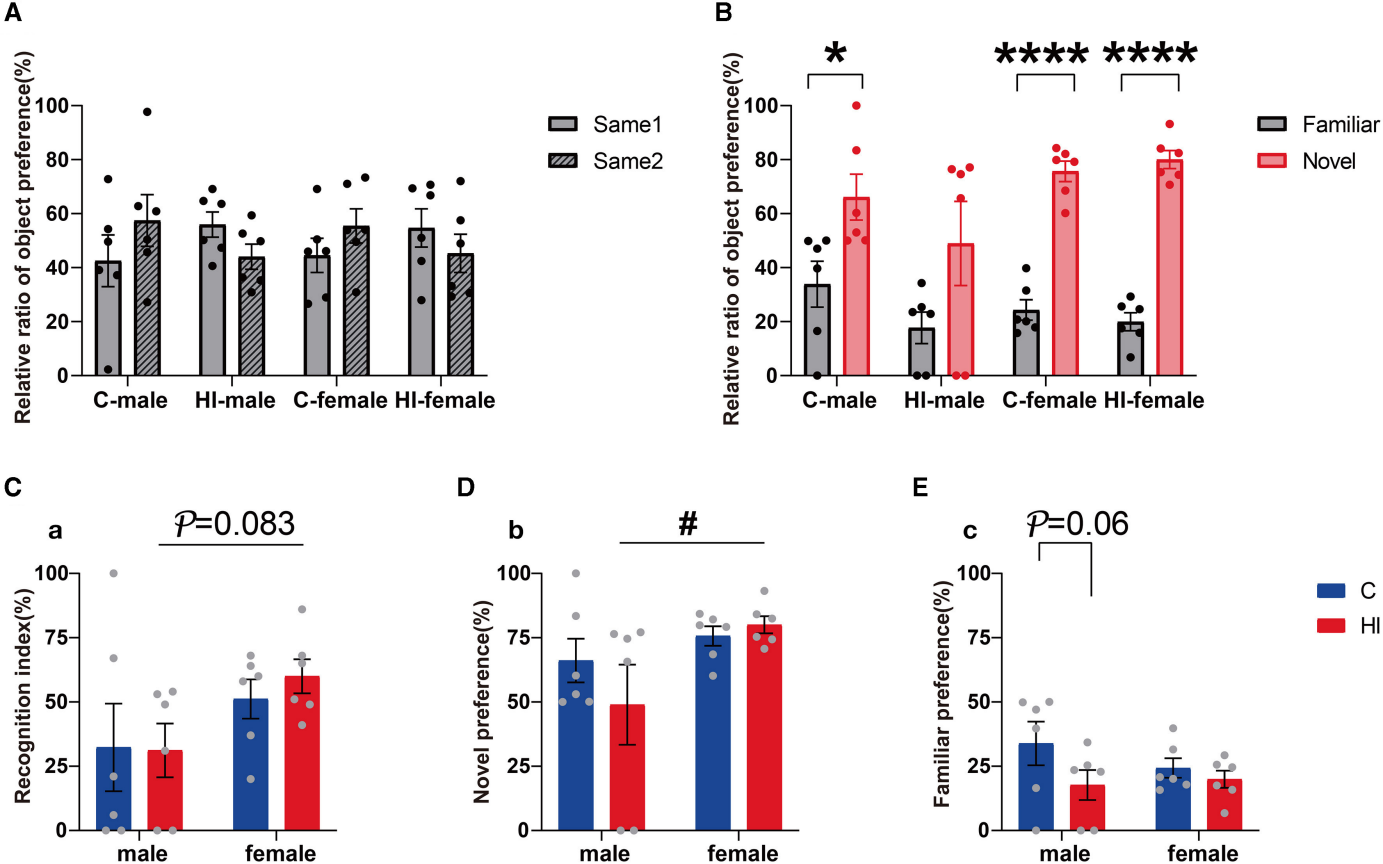
Evaluation of recognition memory ability in PND62 rats. **(A, B)** The relative ratios of object preference for two identical objects (same 1 and same 2) in the training phase and familiar and novel objects (familiar and novel) in the test phase are shown, respectively. **(C)** Recognition index. **(D, E)** The ratios of novel and familiar object preference are shown, respectively. **(A–E)** Data are presented as means ± SEM. *P < 0.05, ****P < 0.0001; ^#^*P* < 0.05. *Familiar vs. novel (Student's *t*-test); ^#^HI-female vs. HI-male (two-way ANOVA followed by the Bonferroni *post-hoc* test) (*n* = 6 per group).

The NOR test was performed at PND 62. In the acquisition trial, we found no significant differences among the groups in the relative preference for exploring two identical objects (Figure 5A). Once a novel stimulus was introduced, preference for the novel object increased significantly in the test for the control males and females and the HI females (Figure 5B; *p* < 0.05, *p* < 0.0001), whereas the HI males showed similar preference (Figure 5B). In the testing phase, compared to the controls, the preference for novel objects and the discrimination index between the novel and familiar objects decreased in HI males and increased in HI females, without statistical significance (Figures 5C, D). However, this effect was statistically significant (Supplementary Table 3; *p* = 0.04, *p* = 0.046) in terms of sex effects. Notably, while both HI males and females showed a reduced preference for familiar objects compared to controls, this trend was more pronounced in HI males (Figure 5E; *p* = 0.06). HI females showed a significant preference for novel objects, while HI males insignificantly distinguished between familiar and novel objects, thus resulting in reduced discrimination rates (Figure 5C). Compared to HI females, HI males showed a tendency toward lower novel object preference and discrimination index (Figures 5C, D; *p* < 0.05, *p* = 0.083), which does not exclude the possibility that HI damage impaired recognition memory in HI males.

Memory-learning ability is associated with the hip. The MRI results showed that MD values in the hip were significantly smaller and larger in HI males than in controls and HI females during the acute (12 h) and subacute (3 d) phases, respectively, thus indicating that HI males developed more rapidly than HI females during the initial phase. Significant separate effects of HI and sex were shown only in the subacute (3 d) phase, supporting the performance of sex differences in HI rats in the NOR test.

#### 3.6.3. Abnormal gait and locomotion coordination after HI injury

**Figure 6:**
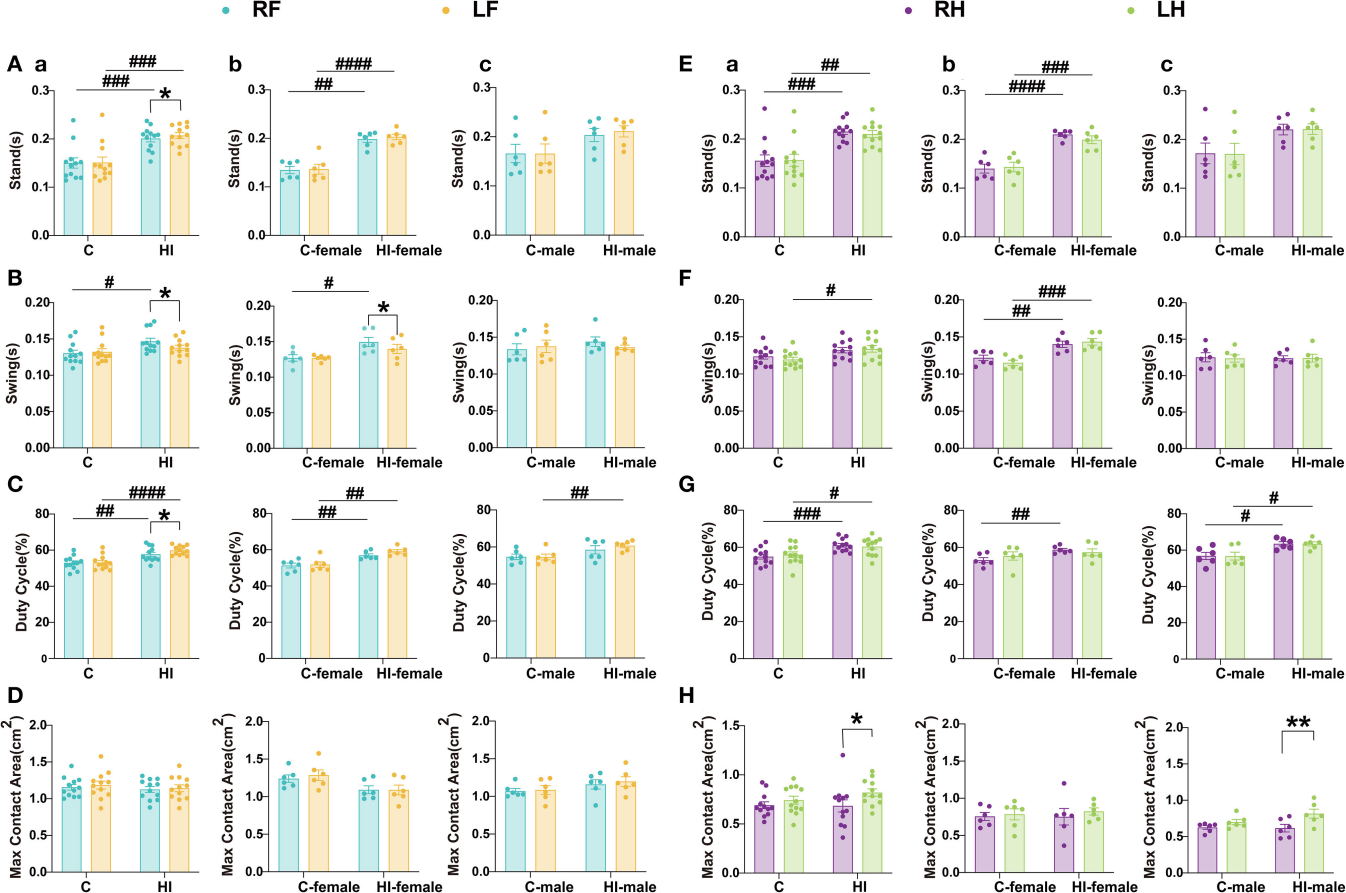
Evaluation of motor function in PND71 rats. **(A–D)** Relevant gait parameters for the front paw. **(E–H)** Relevant gait parameters for the hind paws. Graphs show the standing time **(A, E)**, swing time **(B, F)**, duty cycle **(C, G)**, and maximum contact area **(D, H)** in overall (a), male (b), and female (c), respectively. **(A–H)** Data are presented as means ± SEM. *P < 0.05, **P < 0.01; ^#^*P* < 0.05, ^##^*P* < 0.01, ^###^*P* < 0.001, ^####^*P* < 0.0001. *RF vs. LF, RH vs. LH (paired *t*-test); ^#^HI vs. C, HI-female vs. C-female, HI-male vs. C-male (two-way ANOVA followed by the Bonferroni *post-hoc* test). RF, right fore; RH, right hind; LF, left fore; LH, left hind limbs (*n* = 6 per group).

The CatWalk assessment of motor function after HI injury in rats at P71 revealed long-term deficits in paw-related behavioral parameters, particularly for the limb standing and swinging duration and the maximum contact area (Figure 6). The results showed that the duration (s) of paw contact with the glass plate in HI rats was significantly increased in standing duration compared to controls (*p* < 0.01, *p* < 0.001), but was only statistically significant in females (*p* < 0.01, *p* < 0.001, or *p* < 0.0001) (Figures 6A, E). Interestingly, the swing time of the right front (RF) paw, right hind (RH) paw, and left hind (LH) paw were significantly increased in HI females compared to controls (*p* < 0.05; *p* < 0.01; *p* < 0.001) (Figures 6B, F). In contrast, there was no significant change in swing time in HI males (Figures 6B, F). Additionally, similar non-significant observations were made for the maximum contact area (Figures 6D, H). To further investigate locomotor deficits and determine whether there was any lateralization, we calculated the duty cycle for individual limbs. The results showed that the duty cycle increased significantly in all four limbs in the HI group (*p* < 0.05, *p* < 0.01, *p* < 0.001, or *p* < 0.0001), reaching significance in the RF, left fore (LF), and RH limbs of HI females (all *p* < 0.01), and in the LF, RH, and LH limbs of HI males (*p* < 0.01; *p* < 0.05; *p* < 0.05) (Figures 6C, G).

The paired *t*-tests showed that the maximum contact area of the RH paw was significantly (*p* < 0.05) reduced in HI animals compared to the LH paw, but they were only statistically significant (*p* < 0.01) in HI males (Figure 6H). Compared to the RF paw, we also observed a significant (*p* < 0.05) increase in standing duration and duty cycle and a significant (*p* < 0.05) decrease in swing time for the LF paw in the HI group (Figures 6A–C), with a significant (*p* < 0.05) swing time found only in HI females (Figure 6B).

Overall, the HI-induced sensorimotor function deficits in rats showed differences by sex. Both HI males and females exhibited dynamic gait function deficits (Figures 6C, G), while HI males also exhibited static gait deficits (Figure 6H). This finding could be explained by the MRI scan results. Our study showed that MD, AD, and RD in the sc were significantly greater in HI males than in controls and HI females during the subacute phase (3 d). Meanwhile, MD, AD, RD of the str, and MD of the tha and ec were significantly smaller in HI males than in controls and HI females during the acute phase (12 h). The HI^*^sex interaction effect was significant, indicating that males progressed faster than females after HI injury.

### 3.7. Histological findings

**Figure 7:**
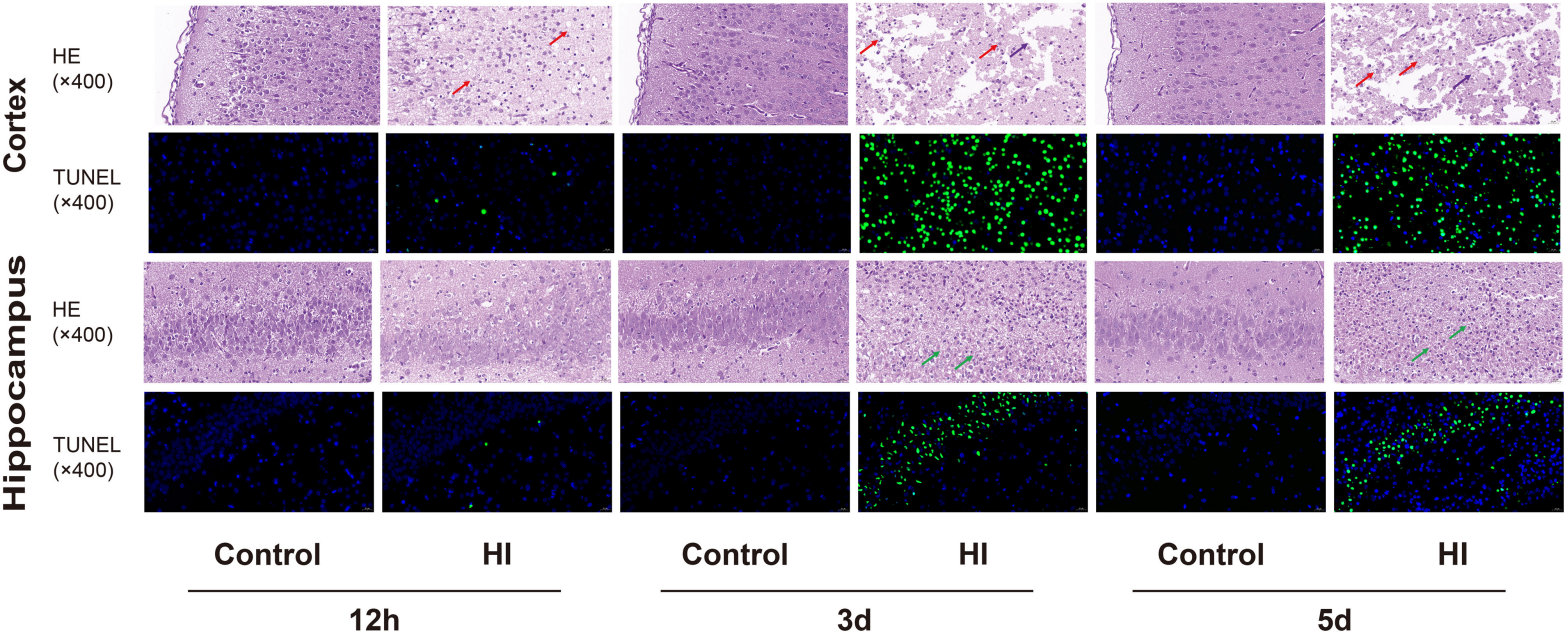
Representative photomicrographs of H&E staining and TUNNEL staining in cortical and hippocampal sections at 12 h, 3 and 5 d (×400). The arrows represent histopathological changes.

H&E and TUNEL staining were performed on the brain sections of the control and HI groups at 12 h and 3 and 5 d, respectively (Figure 7). H&E staining showed that in the acute phase (12 h), the cortex was extensively depigmented and sparsely structured, with more neuronal necrosis visible and the nuclei solidified and deeply stained, fragmented, or lysed and lost (red arrows); the neurons in the hippocampus were slightly irregularly arranged. In the subacute phase (3 and 5 d), the cortex was extensively necrotic and structurally eosinophilic, with an extensive reduction in the number of neurons, visible as large numbers of necrotic cell fragments (red arrows) and rare microglial cell proliferation (purple arrows); and one side of the hippocampus was necrotic and structurally disorganized, with extensive visible fragments of necrotic pyramidal cells (green arrows). Additionally, TUNEL staining showed a gradual increase in the number of apoptotic cells in the cortical and hippocampal regions of the HI group as time progressed.

## 4. Discussion

Here, we investigated the changes in DK and DT parameters in brain tissue over time and the development of complex behaviors, as well as the effect of sex on these parameters. Our findings imply a critical complementary role of DK and DT parameters in the early identification of infants at risk for long-term harm, in which sex played an influential role. These results have the potential to contribute to considerable and long-term improvements in the quality of life of newborns.

Diffusion-weighted MRI, especially DTI, is an effective tool for identifying tissue microstructural changes *in vivo*. However, the Gaussian distribution of diffusion distances inherent to DTI can obscure information about underlying tissue heterogeneity. In contrast, DKI can obtain all of the commonly used DTI diffusion measures plus additional measures associated with DK. DK is a more reliable imaging marker that can provide details on tissue heterogeneity and is less sensitive to certain confounding effects, thus revealing information that MD or FA cannot (Jensen and Helpern, 2010). Our results suggest that MK provides additional information to DT parameters and is sensitive to regional tissue heterogeneity both in the white and gray matter after HI damage. Currently, DK parameters are considered more sensitive than DT parameters in evaluating ischemic lesions, especially MK, which is an index to evaluate the complexity of tissue microstructure and can detect early microstructural changes (Zhu et al., 2019; DiBella et al., 2022). MD reflects the overall level of diffusion resistance and molecular diffusion, but it does not directly reflect the different metabolic conditions within the lesion (Hui et al., 2012a; Li et al., 2019). MK, as the most specific parameter in DKI, can reflect the heterogeneity and complexity of the cellular milieu (Hui et al., 2012a; Lampinen et al., 2021). In other words, the diffusion coefficient only reflects the pathological degree of brain edema, whereas MK reflects the degree of damage to intracellular organelles and cellular structures.

In term infants, HI events result in a different neuropathology pattern from that observed in preterm infants due to the greater vulnerability of gray matter to excitotoxicity at this age (Jensen, 2002). Thus, ischemic injury in term newborns is more typically characterized by gray matter damage, particularly in the cortex, hip, str, and tha, with neuronal damage being a major factor (Fatemi et al., 2009; Volpe, 2009). DTI has been widely used to characterize white matter disease; however, imaging changes in the gray matter microstructure are limited due to gray matter isotropy. MK, provided by DKI, can recognize changes in gray matter. We detected changes over time in DK and DT parameters of specific key structures in HI rats. During the acute phase (12 h), the sensitivity of MK to early changes in brain structures, including the display of deep gray matter, was significantly better than DT parameters. The regions without any effect were mainly focused on the display of DT parameters in the deep gray matter. Falangola et al. (2008) demonstrated that MK is sensitive to changes in gray matter and increases with brain maturation, while FA and MD remain essentially unaltered. Wang et al. (2018) similarly showed longitudinal changes in DKI parameters after traumatic brain injury, and demonstrated that MK detects microstructural changes more sensitively than FA and MD, especially in the gray matter.

Notably, during the acute phase (12 h), the change rate of MK values in the lesions was significantly higher than that of DT parameters, suggesting that MK values demonstrate more complex damage than that demonstrated by DT parameters and that MK can provide more comprehensive diffusion information, reflecting the complexity of hypoxic-ischemic tissue structure more effectively and accurately. Taken together, MK is sensitive to the increase in tissue microstructural complexity induced by HI, which can increase the sensitivity of identifying information at various stages of cerebral ischemia. The MK value, as a more sensitive indicator of damage than DT parameters, may be utilized to characterize the microenvironmental complexity and water molecular heterogeneity in tissues (Hui et al., 2012b; Spampinato et al., 2017; Yin et al., 2018).

Compared to the normal side in the acute phase (12 h), the percentage changes in descending order of MK in different locations were as follows: cc, hip, ic, ec, str, sc, tha, cla, hyp, and mc. This may indicate that when HI in term neonates affects tissues predominantly containing white matter, the microstructural alterations in the tissues are substantially more complex than those in other affected regions with more gray matter. As previously stated, the cc, ic, and ec mainly contain white matter, while less white matter is found in the hip, which contains a mixture of gray and white matter. The str, sc, tha, cla, hyp, and mc contained only gray matter. These findings suggest that the complexity of microstructural alterations in term neonates caused by HI is substantially higher in regions that predominantly comprise white matter than that in other affected regions, except the hip. Although there is no strong evidence to explain why the affected tissue in the hippocampus behaves differently from that in other regions, it may be related to the microstructure of the hip.

In acute brain injury, decreased MD or increased FA is associated with higher cell density or cytotoxic edema, resulting in a smaller extracellular space (Shanmuganathan et al., 2004; Bazarian et al., 2007). Simultaneously, increased MD or decreased FA is associated with cellular membrane breakdown, cell death, tissue cavitation, and vasogenic edema, resulting in increased extracellular space for chronic brain damage (Wieshmann et al., 1999; Cercignani et al., 2001). In several human studies (Bazarian et al., 2007; Niogi et al., 2008) and animal studies (Mac Donald et al., 2007; Immonen et al., 2009), white and gray matter showed decreased FA or increased MD throughout the long-term phase. The pathogenesis of neonatal HIE mainly involves hemodynamic alterations, lactic acid accumulation, oxygen radical accumulation, calcium influx, and excitatory amino acid toxicity, a series of biochemical chain reactions that cause necrosis and apoptosis of neuronal cells. Cerebral hypoxia-ischemia affects ATP synthesis, resulting in reduced Na+-K+-ATPase activity in the cell membrane, which cannot maintain high intracellular and low extracellular osmotic pressure. Large amounts of water molecules and Na+ are transported from the extra to intracellular space, resulting in excessive accumulation of intracellular water and cytotoxic edema. The intercellular space becomes smaller, the diffusion of water molecules becomes restricted, the MK value increases, and the MD, AD, RD, and FA values decrease. Capillary endothelial cells and astrocytes constituting the blood-brain barrier become edematous, disrupting the blood-brain barrier and resulting in vasogenic edema. The influx of large amounts of water molecules and Na+ from the cell into the extracellular space increases interstitial space, thereby alleviating the diffusion restriction of water molecules. The FA and MK values gradually decrease, while the MD, AD, and RD values gradually increase. During this process, the diffusion of water molecules can approach normal, and pseudo-normalization can occur. HE staining showed a gradual increase in necrotic cells, with marked structural disorganization from the acute to subacute phase, while TUNNEL staining showed a gradual increase in apoptotic cells, illustrating the pseudo-normalization of MD, AD, RD, and MK in this process.

Most of the lesions were located in the cortical and subcortical white matter regions of the right cerebral hemisphere due to the redistribution of blood flow to hypermetabolic brain regions during ischemia and hypoxia. The MK value reflects the structural complexity of injured tissue. The pattern of HI injury observed by MK, with a sharp increase in the MK value of the lesion region at 12 h post-injury, demonstrates a dramatic change in the degree of injury between 0 and 12 h, indicating rapid progression of hypoxic and ischemic brain cytotoxic edema. This finding is in agreement with the results of Xiao et al. (2020) and Ma et al. (2022), suggesting a continuous deterioration of the lesion. Then, it gradually decreased over 5 days, approaching baseline levels, but it still remained higher than that in the controls. The degree of change was greater from 12 to 3 d, but it was relatively small from 3 to 5 d, suggesting that vasogenic edema progresses rapidly over 12–3 d, leading to a role for increased extracellular space.

Notably, despite the enlarged extracellular space and the presence of excess fluid due to vasogenic edema 3–5 days after hypoxic ischemia, MK in ischemic tissues remained higher than that in normal tissues, while MD started to pseudo-normalize. Hu et al. (2008) discovered that following cerebrospinal fluid suppression via inversion recovery, MD in GM dropped by 31.3%, whereas MK rose by only 7.6%. Therefore, MK is a more reliable and specific indicator of microstructural alterations than MD, providing microstructural information that is less susceptible to partial volume contamination caused by changes in total brain volume such as vasogenic edema. Another study (Yang et al., 2013) showed that kurtosis metrics were less sensitive to the partial volume of cerebrospinal fluid in cortical gray matter compared to traditional diffusion metrics. The kurtosis metrics may be more specific indicators of tissue microstructure alterations; thus, the observed pseudo-normalization of MD may be attributed solely to vasogenic edema and not to microstructural alterations. In contrast, despite the additional fluid in the ischemic tissue due to vasogenic edema, MK remains specific and sensitive to the underlying microstructural environment, which may indicate that DK could detect specific structural changes that cannot be detected by other techniques during the degradation process. It is important to distinguish the pseudo-normalization of MD from that of MK. During the chronic phase (>5 days), MK began to pseudo-normalize; due to the high tolerance of MK to free fluid contamination, the higher MK 3–5 days after HI may indicate that the ischemic cell membrane remains an important source of diffusion limitation despite the presence of vasogenic edema.

In a longitudinal DK and DT parameter study, we demonstrated injury-specific time-related diffusion changes in HI rats compared to control animals. Then, we conducted a horizontal study, using two-way ANOVA, focusing on the injury-specific sex-related changes in DK and DT parameters across brain regions during the acute and subacute phases of HI. Our results demonstrate that the effect of sex was mainly concentrated in the acute (12 h) and subacute (3 d) periods, while the HI effect was mainly significant in the subacute period (5 d). HI injury showed varying effects on brain structures at all three time points. Notably, the HI effect was not significant for FA values in all of the brain regions during the acute phase (12 h), with similar observations for focal regions. Compared to DT parameters, MK was more sensitive to the main effects of all of the brain structures during the acute (12 h) and subacute (3 d) phases, while effectively complementing the information of DT parameters during the subacute (5 d) phase; this may be caused by the proximity of MK values to the baseline in the subacute phase (5 d), and it is consistent with the phenomenon of complementary inhomogeneous high signal regions in MD, AD, RD, and MK maps in the subacute phase.

Considering various diffusion models, each model has its own limitations. In this study, compared to MK, we found that MD, AD, and RD showed more significant differences between HI males and females. MD, AD, and RD values were significantly smaller and larger in HI males than those in controls and HI females in the acute (12 h) and subacute (3 d) phases, respectively. In the acute phase, the HI^*^sex interaction was mainly significant in the str, tha, and ec, while in the subacute phase, it included the hip, where the separate effect of HI or sex was significant, and the sc, where the HI^*^sex interaction was significant. Although the hip in the acute phase and the ec in the subacute phase had a similar presentation, the HI effect was significant.

Neurological damage is variable in the neonatal HI population, but clear cognitive and behavioral impairments occur as children age. Our study found sex differences mainly in the acute (12 h) and subacute (3 d) periods using a two-way ANOVA with DK and DT parameters at three time points. In this study, we aimed to confirm whether sex differences in acute and subacute MRI findings were significant in an established PND7 HI model (Rice III et al., 1981). While ensuring that the males and females were matched and that the pups were weight controlled, we hypothesized that the pattern of neurological damage would be more severe in HI males than in HI females. Our results support a “protection” of females against adverse outcomes, at least in terms of cortical, str, tha, ec, and hip measurements.

With regard to locomotor behavior in an open field, most studies have reported that HI mice or rats exhibit hyperactivity in spontaneous OFTs (Balduini et al., 2000; McAuliffe et al., 2006; Arteni et al., 2010; Barkhuizen et al., 2019; Sanches et al., 2019). However, OFTs on HI rats have shown inconsistent outcomes, with either males (Barkhuizen et al., 2019) or females (Sanches et al., 2015) showing enhanced locomotor performance. Some anxiety indicators, such as decreased or increased time spent in the central area of the OFT, are present in HI males (Waddell et al., 2016; Borjini et al., 2019); however, increased time spent in the closed arms of the EPM has been primarily observed in HI females (Sanches et al., 2015). Our results showed that HI females spent less time in contact with the wall and more time exploring the unprotected central area, as well as moving faster, covering greater distances, and spending less time at rest than controls and HI males. The EPM test showed that HI animals showed an interesting sex trend in the number of open-arm entries, with HI females having lower levels of anxiety than HI males. Although similar trends were observed for the time spent in both arms and the anxiety index, they were not significant, which may be related to the short duration of the observations. The lack of convincing evidence for sex-specific anxious and exploratory behaviors may stem from the scarcity of sex-comparative research controlling for HI methods, animal age, and brain injury severity (Netto et al., 2017). Our MRI results are consistent with those of Gadian et al., suggesting that the hip and str can be affected by HI (Rademakers et al., 1995; Toft, 1999; Almli et al., 2000; Gadian et al., 2000; Wagner et al., 2002; Mañeru et al., 2003). These structures have been linked to certain cognitive functions including attention and memory, and they are hypothesized to play a role in the pathogenesis of schizophrenia, attention deficit and hyperactivity disorder, and autism (DeLong, 1992; Lou, 1996; Simon, 1999; Dilenge et al., 2001; De Haan et al., 2006). Shen et al. (1991) and McAuliffe et al. (2006) have reported that HI damage to the hip resulted in hyperactivity, while others have shown increased rates of hyperactivity in children with moderate neonatal encephalopathy (NE) (Moster et al., 2002; Marlow et al., 2005) and autism in children with moderate and severe NE (Badawi et al., 2006). According to the performance of MD values in the hip during the acute (12 h) and subacute (3 d) phases and the performance of MD values in the str during the acute phase (12 h), we speculate that neurodevelopmental disorders develop more slowly in HI females than in HI males, which leads to anti-anxiolytic hyperactivity-like behaviors in HI females. HI males showed neither more abnormal motor function nor more anxious traits compared to the controls during adulthood following the OFT and elevated plus maze. However, we found a tendency toward an increase in the proportion of peripheral time for HI males compared to the controls, which may be related to the relatively small sample size of our study. We cannot rule out the possibility of anxiety-autistic-like behavior in HI males. Our research indicates that although HI injury can lead to rats being more active in the OFT test, when exercise requires a higher level of coordination, as in the EPM test, the activity of the rats is diminished, possibly because they reflect anxiety responses under elevated conditions. Thus, in mild anxiogenic or anxiolytic-like conditions such as the OFT, the animals were overactive, showing a hyperexcitable phase, whereas in elevated conditions, such as the EPM test, they displayed decreased exploratory activity, which is consistent with the behavioral responses observed by Lubics et al. (2005).

The NOR test was implemented to assess the effect of HI injury on cognitive functions, notably memory, and the following parameters were considered: preference for two identical objects, assessed during the training phase; discrimination index and preference for familiar and new objects, recorded during the testing phase. Our data clearly indicate that the memory capacity of HI animals was affected in relation to sex. Compared to HI females, HI males showed a lower preference for new objects and discrimination index. Considering the insignificant increase in preference for new objects only in HI males when introduced during the test phase, we cannot rule out the possibility that HI impaired recognition memory performance more severely in HI males. Previous studies have shown that while cognitive and memory impairments are present in all HI animals, HI males are more prone to episodic memory impairment (Pereira et al., 2008; Waddell et al., 2016). Although animal studies on neonatal brain injury rarely report sex-specific outcomes, the clinical literature suggests that females generally have less severe cognitive or memory deficits compared to males (Leversen et al., 2011; Cserjesi et al., 2012). In a meta-analysis (Muntsant et al., 2019), human experiments on performance IQ outcomes and overall IQ revealed a robust female advantage, and animal experiments on the cognitive and behavioral domains showed that HI males had notable behavioral deficits, while the female advantage appeared to be stronger in learning or memory. Indeed, analyses of MRI data in the current study showed the performance of MD values in the hip during the acute (12 h) and subacute (3 d) phases supported this finding, suggesting that HI males developed more rapidly than HI females at the initial phase. In summary, our study suggests that memory function differs across sexes and may be decreased by neonatal brain damage.

The CatWalk evaluation of gaits revealed long-term deficits in the paw following HI injury. Overall, HI females performed better than HI males, which is consistent with a study in PND28 rats (Borjini et al., 2019), in which HI males had more severe sensorimotor dysfunction than HI females. The gait outcome pattern in HI rats appears to be explained by the performance of MD, AD, and RD in sc during the subacute phase (3 d). The sex differences in striatal, thalamic, and external capsule performance of MD during the acute phase (12 h) also appear to contribute to it. The model of neonatal HI in PND7 rats studied by Rice III et al. (1981) mainly damaged the middle cerebral artery regions, including the sc, str, tha, hip, and ec, which were essential for maintaining sensorimotor function in rats. Interestingly, HI injury caused sensorimotor function deficits in several parameters, whereas no significant differences in MD, AD, and RD values between HI and control groups of the same sex were observed in the mc during the subacute phase (3 d), only showing significantly greater values in HI males than in HI females. This might be explained by the exceptional plasticity of the newborn brain, which allows compensatory processes to function, such as the transfer of motor coordination control from injured to non-affected brain areas (Borjini et al., 2019). Taken together, the reporting pattern of rat behavioral outcomes appears to be explained by MRI parameters of specific brain structures.

Nevertheless, the mechanisms behind the sex differences in outcomes are unclear. Some studies suggest that testosterone may worsen injury (Hill et al., 2011) or that estrogen may have a protective effect (Nematipour et al., 2020), while others indicate that sex differences in cell death pathways may favor females (Renolleau et al., 2007; Charriaut-Marlangue et al., 2018; Bonnin et al., 2020). Recently, advanced neuroimaging methods such as magnetic resonance imaging (MRI), diffusion-weighted MRI, and magnetic resonance spectroscopy have been used to quantify brain damage and metabolite abnormalities in HI models at different time points after injury (Zhu et al., 2008; van de Looij et al., 2011; Huang et al., 2016; Xiao et al., 2020; Tabacaru et al., 2023). A previous study showed that PND7 HI males developed more severe nervous brain damage, reflex deficits, hemiplegia, and memory impairment than females (Huang et al., 2016). Moreover, this female advantage was associated with sex differences in short- and long-term MRI. Studies have also shown that long-term cortical loss was greater in male pups than in female pups after PND3 HI, with females being less impaired at PND 25 (van de Looij et al., 2011). Additionally, Huang et al. (2020) indicated that MRI showed significantly less residual brain volume and more severe hemiplegia in males than in females after PND7 HI. This female dominance in both full-term and preterm HI rats may be connected to sex-dependent organelle function during development.

Although rats of both sexes could be used for HI modeling, most previous research has selected male rats to eliminate potential bias related to sex differences (Netto et al., 2017). In our model, HI in the acute (12 h) and subacute (3 d) phases resulted in differences in brain injury levels between males and females on MRI, and we observed that HI females performed better than males in the NOR and CatWalk tasks. Although often overlooked, the clinical literature notes that male infants are more susceptible to perinatal injury than female infants, and also suffer more from long-term behavioral and cognitive abnormalities, including autism, learning disabilities, and cerebral palsy (Hindmarsh et al., 2000; Donders and Hoffman, 2002; Marlow et al., 2005; Johnston and Hagberg, 2007; Kent et al., 2012; Aravamuthan et al., 2020). As with humans, female dominance can be observed in the HI model at different stages of CNS development by MRI and behavioral analysis. It is also important to consider sex interactions when intervening early in life and assessing the outcome of prevention and treatment strategies. Indeed, Barkhuizen et al. (2019) recently found higher mortality and morbidity in HI males than in females and sustained cognitive rescue in HI female rats following the long-term efficacy of multifunctional adult progenitor cells. Pereira et al. (2008) and Tsuji et al. (2010) found sex-specific neuroprotective patterns in rats following environmental reinforcement or rehabilitation training, with females showing the greatest resilience. Interestingly, Muntsant et al. (2019) showed that although HI males performed more poorly on memory tests than HI females, the improvement in HI males was evident with neonatal handling.

In humans, the early use of MRI can provide additional prognostic information on the severity of HI brain injury, and it is readily available in the clinic. In infants, MRI can be used as an auxiliary outcome measure associated with standardized neurological examinations. In rodent trials of potential neuroprotective therapies, MRI results are increasingly used to complement the assessment of prognostic outcomes, including in neonatal HI rat models (Barkovich and Truwit, 1990; Robertson et al., 1999; Kitamura et al., 2011; Wintermark et al., 2011; Tusor et al., 2012; Bosemani et al., 2014; De Vis et al., 2015; Lally et al., 2019; Xiao et al., 2020; Shibasaki et al., 2021; Jeong et al., 2022). In our discussion of DK and DT parameters, MK is more sensitive to early microstructural changes, is highly tolerable, and has the potential to detect white and gray matter alterations, whereas MD is more sensitive in predicting poor neurodevelopmental outcomes and reasonably reflects potential sex differences following HI injury.

There are some limitations to this study. First, to ensure the status of neonatal pups, MRI scans were not performed within 12 h after HI; therefore, no changes in DK and DT parameters were observed in the earliest phase of injury, and MRI scans were not extended to the chronic phase 7 d after HI. Although our results suggest that the trends in DK and DT parameters during the acute (12 h) and subacute (3 and 5 d) phases can be interpreted as being related to cytotoxicity and vasogenic edema, we does not exclude the potential of additional physiological processes that may affect DK. Sexual dimorphism in plasticity, recombination, and developmental compensation may result in alterations to DK and DT parameters in tissue microstructure. Thus, future studies should explore the reorganization mechanisms of the HI models for both sexes. In neonatal HI models, behavioral deficits may not be exclusively attributed to the extent of brain injury, but may be associated with some recombination mechanisms. Future research should focus on exploring the significance of these physiological processes. However, it is evident that DK parameters could be valuable in cases of mild injury, particularly when conventional MRI fails to identify any focal lesions or standard DT parameters can detect minor injury. In addition, some extreme values appeared in some of the behavioral parameter plots, which may be related to our sample size not being large enough. We will improve them in subsequent experiments.

We combined DKI with 12 directions and 3 b values with standard DTI to simulate the standard DKI model for estimating DK and DT parameters; this method was selected by considering the possibility of animal movement under prolonged MRI scanning, the effects of long-term anesthesia on animals, and time constraints on magnet use. Using multiple b values in the DKI protocol increases the accuracy of parameter estimation (Hui et al., 2008; Cheung et al., 2009). Although using only three b-values, with a maximum b-value of 2,000 s/mm^2^, may result in some estimation errors for DKI parameters, such acquisition is considered more practical in clinical scenarios (Jensen and Helpern, 2010). Further, the use of 4.7-T animal MRI is closer to the 3.0-T MRI used clinically, which is more conducive to the translation of animal experimental results into the clinic.

In summary, MK has the potential to characterize different locations of brain tissue affected by HI in full-term neonates. Our results show that the MK shows temporal sensitivity to the monitoring of early microstructural changes. The time point of pseudo-normalizing in MK is delayed compared to MD, AD, and RD. Simultaneously, the observed diffuse characteristic changes provide early evidence of diffuse developmental abnormalities in brain white and gray matter after HI injury, highlighting the potential of kurtosis in detecting white and gray matter alterations. Additionally, DT parameters provide MK with early additional information on potential sex-differentiated effects following HI injury. HI affects anxiety, cognition, and locomotion in a sex-dependent manner, with males being more vulnerable. Given that DT and DK parameters can provide complementary information in many aspects, we suggest that the DKI technology can reveal differences in microstructural alterations at various regions impacted by HI in full-term neonates, and it may be an effective imaging tool for the future early diagnosis, assessment, and monitoring of the neuroprotective and therapeutic effects of neonatal HI brain injury.

## Data availability statement

The original contributions presented in the study are included in the article/Supplementary material, further inquiries can be directed to the corresponding authors.

## Ethics statement

The animal study was approved by Ethics Committee of the Third Affiliated Hospital of Zhengzhou University. The study was conducted in accordance with the local legislation and institutional requirements.

## Author contributions

JB: experimental design, experimental implementation, data acquisition, statistics, and writing. XZhang and XZhao: experiment design and funding provision. All authors contributed to the article and approved the submitted version.
