# Supplementary material for: MR imaging and outcome in neonatal HIBD models are correlated with sex: the value of diffusion tensor MR imaging and diffusion kurtosis MR imaging
Source: Front Neurosci. 2023 Sep 15;17:1234049. doi: 10.3389/fnins.2023.1234049 (PMC10543095; doi:10.3389/fnins.2023.1234049)
Supplement: Supplementary file 1 [file Data_Sheet_1.pdf]

Supplementary Material

MR imaging and outcome in neonatal HIBD models are correlated with sex: the value of diffusion tensor MR imaging and diffusion kurtosis MR imaging

Jieaoxue Bao

*** Correspondence:** Xiaoan Zhang: zxa@zzu.edu.cn

Xin Zhao: zdsfyzx@zzu.edu.cn

TABLE S1 Summary of the statistical results from rm ANOVA of DT/DK parameters in the ROIs after HI injury.

ROI	ANOVA	DK-parameters	DT-parameters			
		MK	FA	MD	AD	RD
mc	Time effect	$F_{2,48}$=10.812, ***p*≤0.0001**	$F_{2,48}$=13.858, ***p*≤0.0001**	$F_{2,48}$=3.51, ***p*=0.038**	$F_{2,48}$=2.321, *p*=0.109	$F_{2,48}$=4.109, ***p*=0.023**
	Time *HI	$F_{2,48}$=1.812, *p*=0.174	$F_{2,48}$=0.879, *p*=0.422	$F_{2,48}$=8.726, ***p*=0.001**	$F_{2,48}$=8.905, ***p*=0.001**	$F_{2,48}$=8.66, ***p*=0.001**
	Time *sex	$F_{2,48}$=1.202, *p*=0.31	$F_{2,48}$=1.068, *p*=0.352	$F_{2,48}$=2.606, *p*=0.084	$F_{2,48}$=2.887, *p*=0.065	$F_{2,48}$=2.403, *p*=0.101
	Time *HI *sex	$F_{2,48}$=1.31, *p*=0.279	$F_{2,48}$=2.09, *p*=0.135	$F_{2,48}$=2.081, *p*=0.136	$F_{2,48}$=1.802, *p*=0.176	$F_{2,48}$=2.243, *p*=0.117
	HI effect	$F_{1,24}$=49.458, ***p*≤0.0001**	$F_{1,24}$=0.001, *p*=0.975	$F_{1,24}$=0.8, *p*=0.38	$F_{1,24}$=0.559, *p*=0.462	$F_{1,24}$=0.932, *p*=0.344
	Sex effect	$F_{1,24}$=9.6, ***p*=0.005**	$F_{1,24}$=3.473, *p*=0.075	$F_{1,24}$=1.28, *p*=0.269	$F_{1,24}$=0.704, *p*=0.41	$F_{1,24}$=1.442, *p*=0.241
	HI *sex	$F_{1,24}$=0.1, *p*=0.755	$F_{1,24}$=0.451, *p*=0.508	$F_{1,24}$=1.032, *p*=0.32	$F_{1,24}$=0.779, *p*=0.386	$F_{1,24}$=1.116, *p*=0.301
sc	Time effect	$F_{2,48}$=11.927, ***p*≤0.0001**	$F_{2,48}$=18.751, ***p*≤0.0001**	$F_{2,48}$=15.052, ***p*≤0.0001**	$F_{2,48}$=11.797, ***p*≤0.0001**	$F_{2,48}$=16.55, ***p*≤0.0001**
	Time *HI	$F_{2,48}$=6.442, ***p*=0.003**	$F_{2,48}$=1.485, *p*=0.237	$F_{2,48}$=25.161, ***p*≤0.0001**	$F_{2,48}$=26.809, ***p*≤0.0001**	$F_{2,48}$=23.721, ***p*≤0.0001**
	Time *sex	$F_{2,48}$=0.169, *p*=0.845	$F_{2,48}$=2.654, *p*=0.081	$F_{2,48}$=2.318, *p*=0.109	$F_{2,48}$=2.256, *p*=0.116	$F_{2,48}$=2.306, *p*=0.111
	Time *HI *sex	$F_{2,48}$=2.175, *p*=0.125	$F_{2,48}$=2.098, *p*=0.134	$F_{2,48}$=3.688, ***p*=0.032**	$F_{2,48}$=3.32, ***p*=0.045**	$F_{2,48}$=3.792, ***p*=0.03**
	HI effect	$F_{1,24}$=78.28, ***p*≤0.0001**	$F_{1,24}$=0.895, *p*=0.353	$F_{1,24}$=5.175, ***p*=0.032**	$F_{1,24}$=3.572, *p*=0.071	$F_{1,24}$=5.834, ***p*=0.024**
	Sex effect	$F_{1,24}$=1.553, *p*=0.225	$F_{1,24}$=5.354, ***p*=0.03**	$F_{1,24}$=1.34, *p*=0.258	$F_{1,24}$=0.507, *p*=0.483	$F_{1,24}$=1.748, *p*=0.199
	HI *sex	$F_{1,24}$=1.081, *p*=0.309	$F_{1,24}$=0.016, *p*=0.9	$F_{1,24}$=0.365, *p*=0.552	$F_{1,24}$=0.436, *p*=0.516	$F_{1,24}$=0.317, *p*=0.578

TABLE S1 (continued)

ROI	ANOVA	DK-parameters	DT-parameters			
		MK	FA	MD	AD	RD
cc	Time effect	$F_{2,23}$=49.120, ***p*≤0.0001**	$F_{2,48}$=1.048, *p*=0.359	$F_{2,48}$=1.88, *p*=0.164	$F_{2,48}$=1.482, *p*=0.237	$F_{2,48}$=2.087, *p*=0.135
	Time *HI	$F_{2,23}$=17.674, ***p*≤0.0001**	$F_{2,48}$=2.582, *p*=0.086	$F_{2,48}$=14.243, ***p*≤0.0001**	$F_{2,48}$=13.509, ***p*≤0.0001**	$F_{2,48}$=14.422, ***p*≤0.0001**
	Time *sex	$F_{2,23}$=1.431, *p*=0.26	$F_{2,48}$=3.418, ***p*=0.041**	$F_{2,48}$=0.836, *p*=0.439	$F_{2,48}$=0.988, *p*=0.38	$F_{2,48}$=0.773, *p*=0.467
	Time *HI *sex	$F_{2,23}$=1.332, *p*=0.284	$F_{2,48}$=0.071, *p*=0.931	$F_{2,48}$=1.201, *p*=0.31	$F_{2,48}$=1.112, *p*=0.337	$F_{2,48}$=1.249, *p*=0.296
	HI effect	$F_{1,24}$=91.648, ***p*≤0.0001**	$F_{1,24}$=3.398, *p*=0.078	$F_{1,24}$=0.749, *p*=0.395	$F_{1,24}$=0.005, *p*=0.943	$F_{1,24}$=1.685, *p*=0.207
	Sex effect	$F_{1,24}$=3.035, *p*=0.094	$F_{1,24}$=2.952, *p*=0.099	$F_{1,24}$=0.105, *p*=0.749	$F_{1,24}$=0.504, *p*=0.485	$F_{1,24}$=0.006, *p*=0.939
	HI *sex	$F_{1,24}$=0.082, *p*=0.777	$F_{1,24}$=0.023, *p*=0.881	$F_{1,24}$=1.492, *p*=0.234	$F_{1,24}$=1.409, *p*=0.247	$F_{1,24}$=1.427, *p*=0.244
ic	Time effect	$F_{2,48}$=15.19, ***p*≤0.0001**	$F_{2,48}$=2.558, *p*=0.088	$F_{2,48}$=1.441, *p*=0.247	$F_{2,48}$=1.034, *p*=0.363	$F_{2,48}$=1.714, *p*=0.191
	Time *HI	$F_{2,48}$=13.478, ***p*≤0.0001**	$F_{2,48}$=0.012, *p*=0.988	$F_{2,48}$=7.638, ***p*=0.001**	$F_{2,48}$=8.048, ***p*=0.001**	$F_{2,48}$=7.075, ***p*=0.002**
	Time *sex	$F_{2,48}$=1.614, *p*=0.21	$F_{2,48}$=3.379, ***p*=0.042**	$F_{2,48}$=0.907, *p*=0.41	$F_{2,48}$=0.513, *p*=0.602	$F_{2,48}$=1.187, *p*=0.314
	Time *HI *sex	$F_{2,48}$=2.265, *p*=0.115	$F_{2,48}$=0.654, *p*=0.524	$F_{2,48}$=0.647, *p*=0.528	$F_{2,48}$=0.302, *p*=0.741	$F_{2,48}$=0.921, *p*=0.405
	HI effect	$F_{1,24}$=41.529, ***p*≤0.0001**	$F_{1,24}$=0.745, *p*=0.397	$F_{1,24}$=0.297, *p*=0.591	$F_{1,24}$=0.027, *p*=0.871	$F_{1,24}$=0.563, *p*=0.46
	Sex effect	$F_{1,24}$=0.103, *p*=0.751	$F_{1,24}$=2.832, *p*=0.105	$F_{1,24}$=1.236, *p*=0.277	$F_{1,24}$=2.512, *p*=0.126	$F_{1,24}$=0.651, *p*=0.428
	HI *sex	$F_{1,24}$=5.575, ***p*=0.027**	$F_{1,24}$=0.093, *p*=0.763	$F_{1,24}$=0.751, *p*=0.395	$F_{1,24}$=1.132, *p*=0.298	$F_{1,24}$=0.525, *p*=0.476

TABLE S1 (continued)

ROI	ANOVA	DK-parameters	DT-parameters			
		MK	FA	MD	AD	RD
ec	Time effect	$F_{2,23}$=14.726, **p≤0.0001**	$F_{2,48}$=8.872, **p=0.001**	$F_{2,48}$=3.241, **p=0.048**	$F_{2,48}$=1.621, p=0.208	$F_{2,48}$=4.304, **p=0.019**
	Time *HI	$F_{2,23}$=10.486, **p=0.001**	$F_{2,48}$=0.277, p=0.759	$F_{2,48}$=11.294, **p≤0.0001**	$F_{2,48}$=11.455, **p≤0.0001**	$F_{2,48}$=10.799, **p≤0.0001**
	Time *sex	$F_{2,23}$=.712, p=0.501	$F_{2,48}$=4.9, **p=0.012**	$F_{2,48}$=1.586, p=0.215	$F_{2,48}$=1.834, p=0.171	$F_{2,48}$=1.518, p=0.23
	Time *HI *sex	$F_{2,23}$=2.662, p=0.091	$F_{2,48}$=0.866, p=0.427	$F_{2,48}$=2.355, p=0.106	$F_{2,48}$=1.939, p=0.155	$F_{2,48}$=2.552, p=0.088
	HI effect	$F_{1,24}$=50.157, **p≤0.0001**	$F_{1,24}$=0.114, p=0.739	$F_{1,24}$=4.773, **p=0.039**	$F_{1,24}$=4.168, p=0.052	$F_{1,24}$=4.769, **p=0.039**
	Sex effect	$F_{1,24}$=0.055, p=0.816	$F_{1,24}$=3.083, p=0.092	$F_{1,24}$=0.009, p=0.925	$F_{1,24}$=0.179, p=0.676	$F_{1,24}$=0.146, p=0.706
	HI *sex	$F_{1,24}$=3.569, p=0.071	$F_{1,24}$=0.175, p=0.679	$F_{1,24}$=0.127, p=0.725	$F_{1,24}$=0.092, p=0.765	$F_{1,24}$=0.137, p=0.714
hip	Time effect	$F_{2,23}$=24.660, **p≤0.0001**	$F_{2,48}$=0.952, p=0.393	$F_{2,23}$=3.378, p=0.052	$F_{2,23}$=3.268, p=0.056	$F_{2,23}$=3.363, p=0.052
	Time *HI	$F_{2,23}$=13.710, **p≤0.0001**	$F_{2,48}$=2.829, p=0.069	$F_{2,23}$=12.144, **p≤0.0001**	$F_{2,23}$=12.670, **p≤0.0001**	$F_{2,23}$=11.639, **p≤0.0001**
	Time *sex	$F_{2,23}$=2.786, p=0.083	$F_{2,48}$=3.483, **p=0.039**	$F_{2,23}$=2.951, p=0.072	$F_{2,23}$=2.050, p=0.152	$F_{2,23}$=3.517, **p=0.046**
	Time *HI *sex	$F_{2,23}$=1.205, p=0.318	$F_{2,48}$=0.311, p=0.734	$F_{2,23}$=2.212, p=0.132	$F_{2,23}$=1.970, p=0.162	$F_{2,23}$=2.277, p=0.125
	HI effect	$F_{1,24}$=42.745, **p≤0.0001**	$F_{1,24}$=9.209, **p=0.006**	$F_{1,24}$=5.675, **p=0.025**	$F_{1,24}$=2.093, p=0.161	$F_{1,24}$=7.962, **p=0.009**
	Sex effect	$F_{1,24}$=0.171, p=0.683	$F_{1,24}$=2.267, p=0.145	$F_{1,24}$=0.023, p=0.882	$F_{1,24}$=0.045, p=0.834	$F_{1,24}$=0.12, p=0.732
	HI *sex	$F_{1,24}$=0.529, p=0.474	$F_{1,24}$=0.07, p=0.794	$F_{1,24}$=0.147, p=0.705	$F_{1,24}$=0.165, p=0.688	$F_{1,24}$=0.125, p=0.727

TABLE S1 (continued)

ROI	ANOVA	DK-parameters	DT-parameters			
		MK	FA	MD	AD	RD
	Time effect	$F_{2,48}$=12.387, **$p \leqslant 0.0001$**	$F_{2,48}$=1.098, p=0.342	$F_{2,48}$=1.322, p=0.276	$F_{2,48}$=1.576, p=0.217	$F_{2,48}$=0.954, p=0.392
	Time *HI	$F_{2,48}$=1.475, p=0.239	$F_{2,48}$=1.119, p=0.335	$F_{2,48}$=0.215, p=0.807	$F_{2,48}$=0.69, p=0.507	$F_{2,48}$=0.197, p=0.822
	Time *sex	$F_{2,48}$=2.637, p=0.082	$F_{2,48}$=0.935, p=0.4	$F_{2,48}$=2.804, p=0.071	$F_{2,48}$=2.052, p=0.14	$F_{2,48}$=2.372, p=0.104
hyp	Time *HI *sex	$F_{2,48}$=1.223, p=0.303	$F_{2,48}$=0.986, p=0.381	$F_{2,48}$=3.105, p=0.054	$F_{2,48}$=4.895, **p=0.012**	$F_{2,48}$=1.395, p=0.258
	HI effect	$F_{1,24}$=13.312, **p=0.001**	$F_{1,24}$=0.097, p=0.758	$F_{1,24}$=0, p=0.995	$F_{1,24}$=0.082, p=0.777	$F_{1,24}$=0.017, p=0.898
	Sex effect	$F_{1,24}$=9.302, **p=0.006**	$F_{1,24}$=0.231, p=0.635	$F_{1,24}$=0.44, p=0.514	$F_{1,24}$=1.132, p=0.298	$F_{1,24}$=0.183, p=0.673
	HI *sex	$F_{1,24}$=0.297, p=0.591	$F_{1,24}$=0.055, p=0.817	$F_{1,24}$=3.887, p=0.06	$F_{1,24}$=4.932, **p=0.036**	$F_{1,24}$=3.08, p=0.092
	Time effect	$F_{2,23}$=13.641, **$p \leqslant 0.0001$**	$F_{2,48}$=4.714, **p=0.014**	$F_{2,48}$=0.158, p=0.854	$F_{2,23}$=.262, p=0.772	$F_{2,48}$=0.283, p=0.755
	Time *HI	$F_{2,23}$=5.936, **p=0.008**	$F_{2,48}$=0.084, p=0.919	$F_{2,48}$=3.426, **p=0.041**	$F_{2,23}$=6.736, **p=0.005**	$F_{2,48}$=3.157, p=0.052
	Time *sex	$F_{2,23}$=2.015, p=0.156	$F_{2,48}$=5.038, **p=0.01**	$F_{2,48}$=1.648, p=0.203	$F_{2,23}$=1.349, p=0.279	$F_{2,48}$=1.75, p=0.185
str	Time *HI *sex	$F_{2,23}$=4.017, **p=0.032**	$F_{2,48}$=0.709, p=0.497	$F_{2,48}$=2.191, p=0.123	$F_{2,23}$=2.526, p=0.102	$F_{2,48}$=2.237, p=0.118
	HI effect	$F_{1,24}$=51.6, **$p \leqslant 0.0001$**	$F_{1,24}$=0.039, p=0.844	$F_{1,24}$=0.302, p=0.588	$F_{1,24}$=0.253, p=0.619	$F_{1,24}$=0.302, p=0.588
	Sex effect	$F_{1,24}$=0.073, p=0.789	$F_{1,24}$=3.361, p=0.079	$F_{1,24}$=0.224, p=0.64	$F_{1,24}$=1.064, p=0.313	$F_{1,24}$=0.018, p=0.895
	HI *sex	$F_{1,24}$=5.943, **p=0.023**	$F_{1,24}$=0.739, p=0.398	$F_{1,24}$=1.442, p=0.242	$F_{1,24}$=1.06, p=0.313	$F_{1,24}$=1.563, p=0.223

TABLE S1 (continued)

ROI	ANOVA	DK-parameters	DT-parameters			
		MK	FA	MD	AD	RD
cla	Time effect	$F_{2,48}$=10.861, **p⩽0.0001**	$F_{2,48}$=1.562, p=0.22	$F_{2,48}$=0.598, p=0.554	$F_{2,48}$=0.669, p=0.517	$F_{2,48}$=0.602, p=0.552
	Time *HI	$F_{2,48}$=10.202, **p⩽0.0001**	$F_{2,48}$=0.282, p=0.756	$F_{2,48}$=3.794, **p=0.03**	$F_{2,48}$=4.83, **p=0.012**	$F_{2,48}$=3.127, p=0.053
	Time *sex	$F_{2,48}$=0.943, p=0.397	$F_{2,48}$=4.783, **p=0.013**	$F_{2,48}$=0.804, p=0.454	$F_{2,48}$=0.326, p=0.723	$F_{2,48}$=1.164, p=0.321
	Time *HI *sex	$F_{2,48}$=2.12, p=0.131	$F_{2,48}$=2.22, p=0.12	$F_{2,48}$=2.194, p=0.123	$F_{2,48}$=1.355, p=0.268	$F_{2,48}$=2.684, p=0.079
	HI effect	$F_{1,24}$=35.244, **p⩽0.0001**	$F_{1,24}$=0.272, p=0.607	$F_{1,24}$=0.362, p=0.553	$F_{1,24}$=0.45, p=0.509	$F_{1,24}$=0.274, p=0.605
	Sex effect	$F_{1,24}$=0.014, p=0.905	$F_{1,24}$=4.059, p=0.055	$F_{1,24}$=0.003, p=0.955	$F_{1,24}$=0.368, p=0.55	$F_{1,24}$=0.069, p=0.795
	HI *sex	$F_{1,24}$=4.998, **p=0.035**	$F_{1,24}$=0.925, p=0.346	$F_{1,24}$=0.59, p=0.45	$F_{1,24}$=0.261, p=0.614	$F_{1,24}$=0.772, p=0.388
tha	Time effect	$F_{2,48}$=6.632, **p=0.003**	$F_{2,48}$=2.233, p=0.118	$F_{2,23}$=1.324, p=0.286	$F_{2,23}$=3.176, p=0.061	$F_{2,23}$=.552, p=0.583
	Time *HI	$F_{2,48}$=7.274, **p=0.002**	$F_{2,48}$=0.215, p=0.808	$F_{2,23}$=2.611, p=0.095	$F_{2,23}$=3.508, **p=0.047**	$F_{2,23}$=1.865, p=0.178
	Time *sex	$F_{2,48}$=1.078, p=0.349	$F_{2,48}$=3.388, **p=0.042**	$F_{2,23}$=1.574, p=0.229	$F_{2,23}$=1.995, p=0.159	$F_{2,23}$=1.784, p=0.19
	Time *HI *sex	$F_{2,48}$=1.881, p=0.164	$F_{2,48}$=0.364, p=0.697	$F_{2,23}$=2.276, p=0.125	$F_{2,23}$=2.936, p=0.073	$F_{2,23}$=1.779, p=0.191
	HI effect	$F_{1,24}$=16.662, **p⩽0.0001**	$F_{1,24}$=0.001, p=0.977	$F_{1,24}$=0.656, p=0.426	$F_{1,24}$=0.751, p=0.395	$F_{1,24}$=0.536, p=0.471
	Sex effect	$F_{1,24}$=0.052, p=0.821	$F_{1,24}$=1.736, p=0.2	$F_{1,24}$=1.514, p=0.23	$F_{1,24}$=2.434, p=0.132	$F_{1,24}$=0.912, p=0.349
	HI *sex	$F_{1,24}$=2.24, p=0.148	$F_{1,24}$=0.479, p=0.496	$F_{1,24}$=2.027, p=0.167	$F_{1,24}$=1.398, p=0.249	$F_{1,24}$=2.261, p=0.146

$P < 0.05$ was considered statistically significant.

Bold values represent significant p-values.

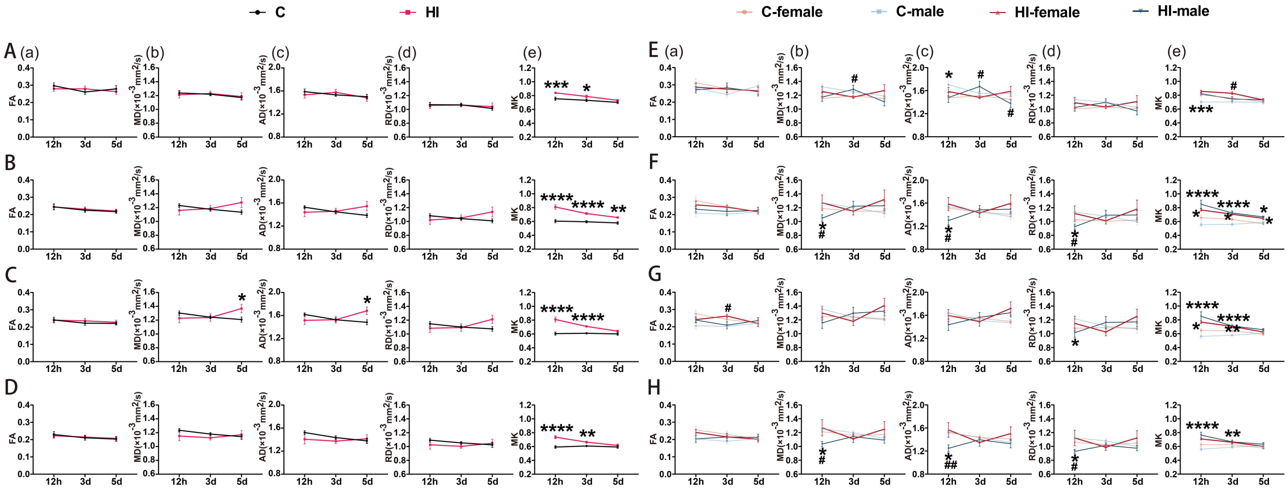

FIGURE S1

Assessment of DT and DK metrics at the level of deep gray matter after HI injury. **(A-D)** Effects of time and HI on DT and DK metrics. **(E-F)** Effect of time, HI and sex on DT and DK metrics. Graphs show FA (a), MD (b), AD (c), RD (d) and MK (e) at the levels of hyp **(A, E)**, str **(B, F)**, cla **(C, G)**, and tha **(D, H)**, respectively. **(A-H)** Data are presented as means ± SEM (two-way rm ANOVA or two-way ANOVA followed by the Bonferroni post hoc test). *$P < 0.05$, **$P < 0.01$, ***$P < 0.001$, ****$P < 0.0001$; #$P < 0.05$, ##$P < 0.01$. *: HI group vs. Control group, HI-female vs. C-female, HI-male vs. C-male; #: HI-female vs. HI-male.

TABLE S2 Summary of the statistical results from two-way ANOVA of DT/DK parameters in the ROIs at three-time points after HI injury.

ROI		ANOVA	DK-parameters	DT-parameters			
			MK	FA	MD	AD	RD
mc	12h	HI effect	$F_{1, 24}$=24.314, **p≤0.0001**	$F_{1, 24}$=0.352, p=0.559	$F_{1, 24}$=3.249, p=0.084	$F_{1, 24}$=3.716, p=0.066	$F_{1, 24}$=3.084, p=0.092
		Sex effect	$F_{1, 24}$=7.181, **p=0.013**	$F_{1, 24}$=1.785, p=0.194	$F_{1, 24}$=1.021, p=0.322	$F_{1, 24}$=1.159, p=0.292	$F_{1, 24}$=0.87, p=0.36
		HI*sex	$F_{1, 24}$=0.658, p=0.425	$F_{1, 24}$=2.332, p=0.14	$F_{1, 24}$=3.137, p=0.089	$F_{1, 24}$=2.375, p=0.136	$F_{1, 24}$=3.542, p=0.072
	3d	HI effect	$F_{1, 24}$=11.177, **p=0.003**	$F_{1, 24}$=0.001, p=0.975	$F_{1, 24}$=0.187, p=0.669	$F_{1, 24}$=0.013, p=0.912	$F_{1, 24}$=0.409, p=0.529
		Sex effect	$F_{1, 24}$=2.023, p=0.168	$F_{1, 24}$=4.567, **p=0.043**	$F_{1, 24}$=7.608, **p=0.011**	$F_{1, 24}$=5.25, **p=0.031**	$F_{1, 24}$=8.081, **p=0.009**
		HI*sex	$F_{1, 24}$=0.005, p=0.946	$F_{1, 24}$=0.079, p=0.782	$F_{1, 24}$=0.926, p=0.345	$F_{1, 24}$=1.102, p=0.304	$F_{1, 24}$=0.771, p=0.389
	5d	HI effect	$F_{1, 24}$=8.505, **p=0.008**	$F_{1, 24}$=0.74, p=0.398	$F_{1, 24}$=9.572, **p=0.005**	$F_{1, 24}$=9.243, **p=0.006**	$F_{1, 24}$=9.456, **p=0.005**
		Sex effect	$F_{1, 24}$=0.536, p=0.471	$F_{1, 24}$=0.922, p=0.347	$F_{1, 24}$=0.66, p=0.425	$F_{1, 24}$=1.215, p=0.281	$F_{1, 24}$=0.415, p=0.525
		HI*sex	$F_{1, 24}$=2.625, p=0.118	$F_{1, 24}$=0.192, p=0.666	$F_{1, 24}$=0.434, p=0.516	$F_{1, 24}$=0.586, p=0.451	$F_{1, 24}$=0.364, p=0.552
sc	12h	HI effect	$F_{1, 24}$=32.5, **p≤0.0001**	$F_{1, 24}$=0.113, p=0.74	$F_{1, 24}$=5.915, **p=0.023**	$F_{1, 24}$=8.59, **p=0.007**	$F_{1, 24}$=4.576, **p=0.043**
		Sex effect	$F_{1, 24}$=0.068, p=0.797	$F_{1, 24}$=5.878, **p=0.023**	$F_{1, 24}$=0.053, p=0.821	$F_{1, 24}$=0.033, p=0.857	$F_{1, 24}$=0.172, p=0.682
		HI*sex	$\mathrm{F}_{1, 24}$=2.962, p=0.098	$\mathrm{F}_{1, 24}$=1.155, p=0.293	$\mathrm{F}_{1, 24}$=1.983, p=0.172	$\mathrm{F}_{1, 24}$=1.408, p=0.247	$\mathrm{F}_{1, 24}$=2.235, p=0.148
	3d	HI effect	$\mathrm{F}_{1, 24}$=40.962, **p≤0.0001**	$\mathrm{F}_{1, 24}$=0.008, p=0.929	$\mathrm{F}_{1, 24}$=0.791, p=0.383	$\mathrm{F}_{1, 24}$=0.219, p=0.644	$\mathrm{F}_{1, 24}$=1.205, p=0.283
		Sex effect	$F_{1, 24}$=1.979, p=0.172	$F_{1, 24}$=5.306, **p=0.03**	$F_{1, 24}$=9.441, **p=0.005**	$F_{1, 24}$=6.8, **p=0.015**	$F_{1, 24}$=9.93, **p=0.004**
		HI*sex	$F_{1, 24}$=0.098, p=0.757	$F_{1, 24}$=0.37, p=0.548	$F_{1, 24}$=6.912, **p=0.015**	$F_{1, 24}$=7.314, **p=0.012**	$F_{1, 24}$=5.965, **p=0.022**
	5d	HI effect	$F_{1, 24}$=7.179, **p=0.013**	$F_{1, 24}$=4.581, **p=0.043**	$F_{1, 24}$=29.023, **p≤0.0001**	$F_{1, 24}$=30.036, **p≤0.0001**	$F_{1, 24}$=27.611, **p≤0.0001**
		Sex effect	$F_{1, 24}$=0.523, p=0.477	$F_{1, 24}$=0.258, p=0.616	$F_{1, 24}$=0.029, p=0.867	$F_{1, 24}$=0.148, p=0.704	$F_{1, 24}$=0.003, p=0.955
		HI*sex	$F_{1, 24}$=0.8, p=0.38	$F_{1, 24}$=0.903, p=0.352	$F_{1, 24}$=0.289, p=0.596	$F_{1, 24}$=0.113, p=0.74	$F_{1, 24}$=0.399, p=0.534

TABLE S2 (continued)

ROI		ANOVA	DK-parameters	DT-parameters			
			MK	FA	MD	AD	RD
cc	12h	HI effect	$F_{1,24}$=43.95, ***p*≤0.0001**	$F_{1,24}$=0.096, *p*=0.76	$F_{1,24}$=5.762, ***p*=0.024**	$F_{1,24}$=7.221, ***p*=0.013**	$F_{1,24}$=4.803, ***p*=0.038**
		Sex effect	$F_{1,24}$=1.132, *p*=0.298	$F_{1,24}$=5.859, ***p*=0.023**	$F_{1,24}$=0.682, *p*=0.417	$F_{1,24}$=1.407, *p*=0.247	$F_{1,24}$=0.33, *p*=0.571
		HI*sex	$F_{1,24}$=0.337, *p*=0.567	$F_{1,24}$=0.067, *p*=0.798	$F_{1,24}$=2.801, *p*=0.107	$F_{1,24}$=2.492, *p*=0.128	$F_{1,24}$=2.941, *p*=0.099
	3d	HI effect	$F_{1,24}$=66.358, ***p*≤0.0001**	$F_{1,24}$=2.941, *p*=0.099	$F_{1,24}$=0.553, *p*=0.464	$F_{1,24}$=0.089, *p*=0.768	$F_{1,24}$=1.875, *p*=0.184
		Sex effect	$F_{1,24}$=4.298, ***p*=0.049**	$F_{1,24}$=3.258, *p*=0.084	$F_{1,24}$=1.888, *p*=0.182	$F_{1,24}$=0.511, *p*=0.482	$F_{1,24}$=2.842, *p*=0.105
		HI*sex	$F_{1,24}$=0.293, *p*=0.593	$F_{1,24}$=0.04, *p*=0.843	$F_{1,24}$=0.189, *p*=0.668	$F_{1,24}$=0.134, *p*=0.717	$F_{1,24}$=0.188, *p*=0.668
	5d	HI effect	$F_{1,24}$=14.426, ***p*=0.001**	$F_{1,24}$=6.563, ***p*=0.017**	$F_{1,24}$=12.714, ***p*=0.002**	$F_{1,24}$=10.255, ***p*=0.004**	$F_{1,24}$=13.79, ***p*=0.001**
		Sex effect	$F_{1,24}$=0.138, *p*=0.714	$F_{1,24}$=0, *p*=0.984	$F_{1,24}$=0.097, *p*=0.758	$F_{1,24}$=0.108, *p*=0.745	$F_{1,24}$=0.087, *p*=0.77
		HI*sex	$F_{1,24}$=0.818, *p*=0.375	$F_{1,24}$=0.008, *p*=0.931	$F_{1,24}$=0.032, *p*=0.859	$F_{1,24}$=0.04, *p*=0.843	$F_{1,24}$=0.025, *p*=0.876
ic	12h	HI effect	$F_{1,24}$=37.716, ***p*≤0.0001**	$F_{1,24}$=0.372, *p*=0.548	$F_{1,24}$=3.714, *p*=0.066	$F_{1,24}$=5.287, ***p*=0.03**	$F_{1,24}$=2.782, *p*=0.108
		Sex effect	$F_{1,24}$=0.247, *p*=0.623	$F_{1,24}$=4.057, *p*=0.055	$F_{1,24}$=0.98, *p*=0.332	$F_{1,24}$=1.893, *p*=0.182	$F_{1,24}$=0.533, *p*=0.473
		HI*sex	$F_{1,24}$=5.464, ***p*=0.028**	$F_{1,24}$=0.165, *p*=0.688	$F_{1,24}$=1.545, *p*=0.226	$F_{1,24}$=1.108, *p*=0.303	$F_{1,24}$=1.742, *p*=0.199
	3d	HI effect	$F_{1,24}$=37.15, ***p*≤0.0001**	$F_{1,24}$=0.642, *p*=0.431	$F_{1,24}$=0.203, *p*=0.656	$F_{1,24}$=0.004, *p*=0.951	$F_{1,24}$=0.485, *p*=0.493
		Sex effect	$F_{1,24}$=2.641, *p*=0.117	$F_{1,24}$=4.733, ***p*=0.04**	$F_{1,24}$=0.043, *p*=0.837	$F_{1,24}$=0.145, *p*=0.707	$F_{1,24}$=0.326, *p*=0.573
		HI*sex	$F_{1,24}$=5.495, ***p*=0.028**	$F_{1,24}$=0.164, *p*=0.689	$F_{1,24}$=0.015, *p*=0.903	$F_{1,24}$=0.035, *p*=0.852	$F_{1,24}$=0.098, *p*=0.757
	5d	HI effect	$F_{1,24}$=2.398, *p*=0.135	$F_{1,24}$=0.392, *p*=0.537	$F_{1,24}$=6.37, ***p*=0.019**	$F_{1,24}$=6.22, ***p*=0.02**	$F_{1,24}$=6.248, ***p*=0.02**
		Sex effect	$F_{1,24}$=1.266, *p*=0.272	$F_{1,24}$=0.099, *p*=0.755	$F_{1,24}$=1.664, *p*=0.209	$F_{1,24}$=1.743, *p*=0.199	$F_{1,24}$=1.536, *p*=0.227
		HI*sex	$F_{1,24}$=0.14, *p*=0.711	$F_{1,24}$=0.64, *p*=0.432	$F_{1,24}$=0.283, *p*=0.6	$F_{1,24}$=0.661, *p*=0.424	$F_{1,24}$=0.123, *p*=0.729

TABLE S2 (continued)

ROI		ANOVA	DK-parameters	DT-parameters			
			MK	FA	MD	AD	RD
ec	12h	HI effect	$F_{1,24}$=26.059, ***p*≤0.0001**	$F_{1,24}$=0.013, *p*=0.909	$F_{1,24}$=4.146, *p*=0.053	$F_{1,24}$=4.626, ***p*=0.042**	$F_{1,24}$=3.539, *p*=0.072
		Sex effect	$F_{1,24}$=0.164, *p*=0.689	$F_{1,24}$=6.028, ***p*=0.022**	$F_{1,24}$=1.07, *p*=0.311	$F_{1,24}$=3.073, *p*=0.092	$F_{1,24}$=0.311, *p*=0.582
		HI*sex	$F_{1,24}$=3.097, *p*=0.091	$F_{1,24}$=0.919, *p*=0.347	$F_{1,24}$=4.307, ***p*=0.049**	$F_{1,24}$=3.121, *p*=0.09	$F_{1,24}$=4.751, ***p*=0.039**
	3d	HI effect	$F_{1,24}$=28.275, ***p*≤0.0001**	$F_{1,24}$=0.075, *p*=0.787	$F_{1,24}$=4.698, ***p*=0.04**	$F_{1,24}$=3.72, *p*=0.066	$F_{1,24}$=4.894, ***p*=0.037**
		Sex effect	$F_{1,24}$=0.679, *p*=0.418	$F_{1,24}$=3.538, *p*=0.072	$F_{1,24}$=2.933, *p*=0.1	$F_{1,24}$=1.195, *p*=0.285	$F_{1,24}$=4.015, *p*=0.057
		HI*sex	$F_{1,24}$=1.836, *p*=0.188	$F_{1,24}$=0.048, *p*=0.829	$F_{1,24}$=1.415, *p*=0.246	$F_{1,24}$=1.284, *p*=0.268	$F_{1,24}$=1.34, *p*=0.258
	5d	HI effect	$F_{1,24}$=10.789, ***p*=0.003**	$F_{1,24}$=0.575, *p*=0.456	$F_{1,24}$=11.861, ***p*=0.002**	$F_{1,24}$=12.563, ***p*=0.002**	$F_{1,24}$=11.227, ***p*=0.003**
		Sex effect	$F_{1,24}$=0.42, *p*=0.523	$F_{1,24}$=0.28, *p*=0.602	$F_{1,24}$=0.042, *p*=0.839	$F_{1,24}$=0.025, *p*=0.875	$F_{1,24}$=0.051, *p*=0.824
		HI*sex	$F_{1,24}$=0.002, *p*=0.964	$F_{1,24}$=0.122, *p*=0.73	$F_{1,24}$=0.015, *p*=0.902	$F_{1,24}$=0, *p*=0.986	$F_{1,24}$=0.035, *p*=0.853
hip	12h	HI effect	$F_{1,24}$=22.288, ***p*≤0.0001**	$F_{1,24}$=0.863, *p*=0.362	$F_{1,24}$=6.394, ***p*=0.018**	$F_{1,24}$=8.676, ***p*=0.007**	$F_{1,24}$=4.912, ***p*=0.036**
		Sex effect	$F_{1,24}$=0.115, *p*=0.737	$F_{1,24}$=3.483, *p*=0.074	$F_{1,24}$=1.005, *p*=0.326	$F_{1,24}$=1.633, *p*=0.214	$F_{1,24}$=0.659, *p*=0.425
		HI*sex	$F_{1,24}$=1.621, *p*=0.215	$F_{1,24}$=0.484, *p*=0.493	$F_{1,24}$=3.76, *p*=0.064	$F_{1,24}$=3.25, *p*=0.084	$F_{1,24}$=3.913, *p*=0.06
	3d	HI effect	$F_{1,24}$=29.212, ***p*≤0.0001**	$F_{1,24}$=7.286, ***p*=0.013**	$F_{1,24}$=6.841, ***p*=0.015**	$F_{1,24}$=2.594, *p*=0.12	$F_{1,24}$=9.485, ***p*=0.005**
		Sex effect	$F_{1,24}$=5.745, ***p*=0.025**	$F_{1,24}$=4.141, *p*=0.053	$F_{1,24}$=4.614, ***p*=0.042**	$F_{1,24}$=2.601, *p*=0.12	$F_{1,24}$=5.589, ***p*=0.027**
		HI*sex	$F_{1,24}$=0.062, *p*=0.806	$F_{1,24}$=0, *p*=0.995	$F_{1,24}$=1.764, *p*=0.197	$F_{1,24}$=1.571, *p*=0.222	$F_{1,24}$=1.704, *p*=0.204
	5d	HI effect	$F_{1,24}$=1.234, *p*=0.278	$F_{1,24}$=12.564, ***p*=0.002**	$F_{1,24}$=19.14, ***p*≤0.0001**	$F_{1,24}$=17.671, ***p*≤0.0001**	$F_{1,24}$=19.614, ***p*≤0.0001**
		Sex effect	$F_{1,24}$=0.169, *p*=0.685	$F_{1,24}$=0.231, *p*=0.635	$F_{1,24}$=0.022, *p*=0.883	$F_{1,24}$=0.018, *p*=0.895	$F_{1,24}$=0.023, *p*=0.881
		HI*sex	$F_{1,24}$=0.612, *p*=0.442	$F_{1,24}$=0.005, *p*=0.943	$F_{1,24}$=0.089, *p*=0.768	$F_{1,24}$=0.089, *p*=0.769	$F_{1,24}$=0.088, *p*=0.77

TABLE S2 (continued)

ROI		ANOVA	DK-parameters	DT-parameters			
			MK	FA	MD	AD	RD
hyp	12h	HI effect	$F_{1,24}$=13.204, ***p*=0.001**	$F_{1,24}$=0.669, *p*=0.421	$F_{1,24}$=0.204, *p*=0.655	$F_{1,24}$=0.738, *p*=0.399	$F_{1,24}$=0.044, *p*=0.836
		Sex effect	$F_{1,24}$=8.775, ***p*=0.007**	$F_{1,24}$=1.232, *p*=0.278	$F_{1,24}$=0.62, *p*=0.439	$F_{1,24}$=0.86, *p*=0.363	$F_{1,24}$=0.392, *p*=0.537
		HI*sex	$F_{1,24}$=2.273, *p*=0.145	$F_{1,24}$=0.182, *p*=0.673	$F_{1,24}$=5.155, ***p*=0.032**	$F_{1,24}$=6.214, ***p*=0.02**	$F_{1,24}$=3.514, *p*=0.073
	3d	HI effect	$F_{1,24}$=4.852, ***p*=0.037**	$F_{1,24}$=0.724, *p*=0.403	$F_{1,24}$=0.084, *p*=0.775	$F_{1,24}$=0.674, *p*=0.42	$F_{1,24}$=0.002, *p*=0.969
		Sex effect	$F_{1,24}$=7.27, ***p*=0.013**	$F_{1,24}$=0.305, *p*=0.586	$F_{1,24}$=6.295, ***p*=0.019**	$F_{1,24}$=4.533, ***p*=0.044**	$F_{1,24}$=4.778, ***p*=0.039**
		HI*sex	$F_{1,24}$=0.324, *p*=0.575	$F_{1,24}$=0.827, *p*=0.372	$F_{1,24}$=0.588, *p*=0.451	$F_{1,24}$=1.922, *p*=0.178	$F_{1,24}$=0.008, *p*=0.929
	5d	HI effect	$F_{1,24}$=1.582, *p*=0.221	$F_{1,24}$=0.417, *p*=0.525	$F_{1,24}$=0.058, *p*=0.811	$F_{1,24}$=0.083, *p*=0.776	$F_{1,24}$=0.196, *p*=0.662
		Sex effect	$F_{1,24}$=0.046, *p*=0.832	$F_{1,24}$=0.253, *p*=0.619	$F_{1,24}$=1.163, *p*=0.292	$F_{1,24}$=0.748, *p*=0.396	$F_{1,24}$=1.029, *p*=0.32
		HI*sex	$F_{1,24}$=0.039, *p*=0.845	$F_{1,24}$=0.498, *p*=0.487	$F_{1,24}$=2.963, *p*=0.098	$F_{1,24}$=5.569, ***p*=0.027**	$F_{1,24}$=1.789, *p*=0.194
str	12h	HI effect	$F_{1,24}$=29.529, ***p*≤0.0001**	$F_{1,24}$=0.002, *p*=0.967	$F_{1,24}$=1.106, *p*=0.303	$F_{1,24}$=1.375, *p*=0.252	$F_{1,24}$=0.911, *p*=0.349
		Sex effect	$F_{1,24}$=0.057, *p*=0.813	$F_{1,24}$=6.246, ***p*=0.02**	$F_{1,24}$=1.096, *p*=0.306	$F_{1,24}$=3.079, *p*=0.092	$F_{1,24}$=0.385, *p*=0.541
		HI*sex	$F_{1,24}$=5.915, ***p*=0.023**	$F_{1,24}$=1.463, *p*=0.238	$F_{1,24}$=5.244, ***p*=0.031**	$F_{1,24}$=4.473, ***p*=0.045**	$F_{1,24}$=5.331, ***p*=0.03**
	3d	HI effect	$F_{1,24}$=32.807, ***p*≤0.0001**	$F_{1,24}$=0.126, *p*=0.725	$F_{1,24}$=0.044, *p*=0.835	$F_{1,24}$=0.024, *p*=0.879	$F_{1,24}$=0.055, *p*=0.816
		Sex effect	$F_{1,24}$=1.706, *p*=0.204	$F_{1,24}$=3.93, *p*=0.059	$F_{1,24}$=1.644, *p*=0.212	$F_{1,24}$=0.303, *p*=0.587	$F_{1,24}$=2.866, *p*=0.103
		HI*sex	$F_{1,24}$=4.771, ***p*=0.039**	$F_{1,24}$=0.318, *p*=0.578	$F_{1,24}$=0.042, *p*=0.839	$F_{1,24}$=0.104, *p*=0.75	$F_{1,24}$=0.013, *p*=0.911
	5d	HI effect	$F_{1,24}$=11.805, ***p*=0.002**	$F_{1,24}$=0.046, *p*=0.832	$F_{1,24}$=2.885, *p*=0.102	$F_{1,24}$=3.117, *p*=0.09	$F_{1,24}$=2.653, *p*=0.116
		Sex effect	$F_{1,24}$=0.837, *p*=0.369	$F_{1,24}$=0.051, *p*=0.823	$F_{1,24}$=0.541, *p*=0.469	$F_{1,24}$=0.61, *p*=0.442	$F_{1,24}$=0.477, *p*=0.496
		HI*sex	$F_{1,24}$=0, *p*=0.99	$F_{1,24}$=0.065, *p*=0.801	$F_{1,24}$=0.142, *p*=0.71	$F_{1,24}$=0.109, *p*=0.744	$F_{1,24}$=0.156, *p*=0.696

TABLE S2 (continued)

ROI		ANOVA	DK-parameters	DT-parameters			
			MK	FA	MD	AD	RD
cla	12h	HI effect	$F_{1,24}$=26.832, ***p*≤0.0001**	$F_{1,24}$=0.002, *p*=0.961	$F_{1,24}$=1.182, *p*=0.288	$F_{1,24}$=1.78, *p*=0.195	$F_{1,24}$=0.91, *p*=0.35
		Sex effect	$F_{1,24}$=0.001, *p*=0.982	$F_{1,24}$=4.239, *p*=0.051	$F_{1,24}$=0.072, *p*=0.791	$F_{1,24}$=0.538, *p*=0.471	$F_{1,24}$=0, *p*=0.993
		HI*sex	$F_{1,24}$=4.622, ***p*=0.042**	$F_{1,24}$=3.592, *p*=0.07	$F_{1,24}$=3.268, *p*=0.083	$F_{1,24}$=1.943, *p*=0.176	$F_{1,24}$=4.034, *p*=0.056
	3d	HI effect	$F_{1,24}$=45.374, ***p*≤0.0001**	$F_{1,24}$=0.677, *p*=0.419	$F_{1,24}$=0.007, *p*=0.932	$F_{1,24}$=0, *p*=0.997	$F_{1,24}$=0.018, *p*=0.895
		Sex effect	$F_{1,24}$=3.728, *p*=0.065	$F_{1,24}$=8.345, ***p*=0.008**	$F_{1,24}$=1.091, *p*=0.307	$F_{1,24}$=0.06, *p*=0.809	$F_{1,24}$=2.192, *p*=0.152
		HI*sex	$F_{1,24}$=5.338, ***p*=0.03**	$F_{1,24}$=0.156, *p*=0.696	$F_{1,24}$=1.035, *p*=0.319	$F_{1,24}$=0.711, *p*=0.407	$F_{1,24}$=1.164, *p*=0.291
	5d	HI effect	$F_{1,24}$=2.216, *p*=0.15	$F_{1,24}$=0.188, *p*=0.669	$F_{1,24}$=4.749, ***p*=0.039**	$F_{1,24}$=6.066, ***p*=0.021**	$F_{1,24}$=3.855, *p*=0.061
		Sex effect	$F_{1,24}$=0.707, *p*=0.409	$F_{1,24}$=0.289, *p*=0.596	$F_{1,24}$=0.432, *p*=0.517	$F_{1,24}$=0.365, *p*=0.551	$F_{1,24}$=0.424, *p*=0.521
		HI*sex	$F_{1,24}$=0.152, *p*=0.7	$F_{1,24}$=0.317, *p*=0.579	$F_{1,24}$=0.202, *p*=0.657	$F_{1,24}$=0.092, *p*=0.764	$F_{1,24}$=0.25, *p*=0.622
tha	12h	HI effect	$F_{1,24}$=23.878, ***p*≤0.0001**	$F_{1,24}$=0.117, *p*=0.735	$F_{1,24}$=1.543, *p*=0.226	$F_{1,24}$=2.141, *p*=0.156	$F_{1,24}$=1.101, *p*=0.304
		Sex effect	$F_{1,24}$=0.03, *p*=0.865	$F_{1,24}$=4.743, ***p*=0.039**	$F_{1,24}$=2.079, *p*=0.162	$F_{1,24}$=4.126, *p*=0.053	$F_{1,24}$=1.026, *p*=0.321
		HI*sex	$F_{1,24}$=4.415, ***p*=0.046**	$F_{1,24}$=0.123, *p*=0.729	$F_{1,24}$=4.284, ***p*=0.049**	$F_{1,24}$=4.061, *p*=0.055	$F_{1,24}$=4.04, *p*=0.056
	3d	HI effect	$F_{1,24}$=9.652, ***p*=0.005**	$F_{1,24}$=0.074, *p*=0.788	$F_{1,24}$=1.941, *p*=0.176	$F_{1,24}$=1.349, *p*=0.257	$F_{1,24}$=2.057, *p*=0.164
		Sex effect	$F_{1,24}$=0.595, *p*=0.448	$F_{1,24}$=1.45, *p*=0.24	$F_{1,24}$=0.866, *p*=0.361	$F_{1,24}$=0.206, *p*=0.654	$F_{1,24}$=1.452, *p*=0.24
		HI*sex	$F_{1,24}$=1.284, *p*=0.268	$F_{1,24}$=1.394, *p*=0.249	$F_{1,24}$=0.002, *p*=0.962	$F_{1,24}$=0.181, *p*=0.674	$F_{1,24}$=0.164, *p*=0.689
	5d	HI effect	$F_{1,24}$=0.591, *p*=0.449	$F_{1,24}$=0.042, *p*=0.84	$F_{1,24}$=0.146, *p*=0.706	$F_{1,24}$=0.18, *p*=0.675	$F_{1,24}$=0.118, *p*=0.735
		Sex effect	$F_{1,24}$=1.133, *p*=0.298	$F_{1,24}$=0.129, *p*=0.723	$F_{1,24}$=2.183, *p*=0.153	$F_{1,24}$=2.044, *p*=0.166	$F_{1,24}$=2.128, *p*=0.158
		HI*sex	$F_{1,24}$=0.001, *p*=0.982	$F_{1,24}$=0.04, *p*=0.844	$F_{1,24}$=0.531, *p*=0.473	$F_{1,24}$=0.471, *p*=0.499	$F_{1,24}$=0.534, *p*=0.472

$P < 0.05$ was considered statistically significant.

Bold values represent significant p-values.

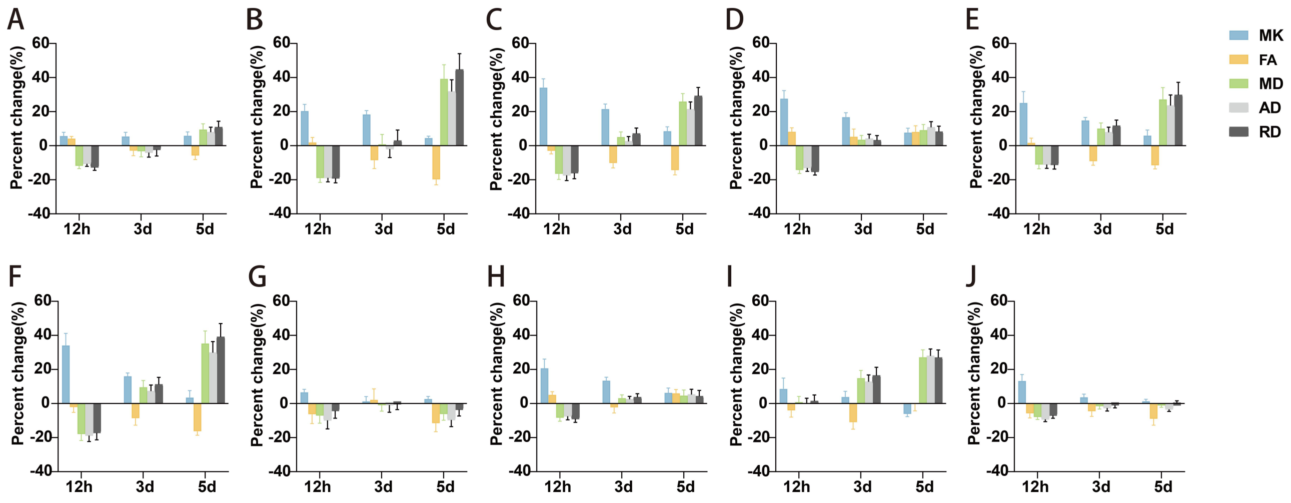

FIGURE S2

Percentage changes of DT and DK metrics for ROIs at 12 h, 3 d, and 5 d after HI injury. **(A)** mc. **(B)** sc. **(C)** cc. **(D)** ic. **(E)** ec. **(F)** hip. **(G)** hyp. **(H)** str. **(I)** cla. and **(J)** tha. Data are presented as means ± SEM.

TABLE S3 Summary of statistical results of the two-way ANOVA for the OFT/EPM/NOR/ test.

NOR	Recognition index(%)	Novel preference(%)	Familiar preference(%)			
HI effect	$F_{1, 20}$=0.117<br>p=0.735	$F_{1, 20}$=0.48<br>p=0.496	$F_{1, 20}$=3.192<br>p=0.089			
Sex effect	$F_{1, 20}$=4.537<br>**p=0.046**	$F_{1, 20}$=4.832<br>**p=0.04**	$F_{1, 20}$=0.398<br>p=0.535			
HI*sex	$F_{1, 20}$=0.2<br>p=0.66	$F_{1, 20}$=1.355<br>p=0.258	$F_{1, 20}$=1.061<br>p=0.315			
EPM	Open arm : time	Close arm : time	Open arm : entries	anxiety index(%)		
HI effect	$F_{1, 20}$=2.055<br>p=0.167	$F_{1, 20}$=0.486<br>p=0.494	$F_{1, 20}$=4.707<br>**p=0.042**	$F_{1, 20}$=2.378<br>p=0.139		
Sex effect	$F_{1, 20}$=1.425<br>p=0.247	$F_{1, 20}$=0.74<br>p=0.4	$F_{1, 20}$=3.664<br>p=0.07	$F_{1, 20}$=1.627<br>p=0.217		
HI*sex	$F_{1, 20}$=0.078<br>p=0.783	$F_{1, 20}$=1.309<br>p=0.266	$F_{1, 20}$=1.971<br>p=0.176	$F_{1, 20}$=0.232<br>p=0.635		
OFT	Distance	Periphery distance	Center distance	Mean speed	Periphery average speed	Center average speed
HI effect	$F_{1, 20}$=5.01<br>**p=0.037**	$F_{1, 20}$=4.11<br>p=0.056	$F_{1, 20}$=3.342<br>p=0.083	$F_{1, 20}$=4.945<br>**p=0.038**	$F_{1, 20}$=4.578<br>**p=0.045**	$F_{1, 20}$=7.077<br>**p=0.015**
Sex effect	$F_{1, 20}$=13.823<br>**p=0.001**	$F_{1, 20}$=10.826<br>**p=0.004**	$F_{1, 20}$=13.679<br>**p=0.001**	$F_{1, 20}$=13.774<br>**p=0.001**	$F_{1, 20}$=12.607<br>**p=0.002**	$F_{1, 20}$=1.701<br>p=0.207
HI*sex	$F_{1, 20}$=4.543<br>**p=0.046**	$F_{1, 20}$=3.669<br>p=0.07	$F_{1, 20}$=3.504<br>p=0.076	$F_{1, 20}$=4.544<br>**p=0.046**	$F_{1, 20}$=4.255<br>p=0.052	$F_{1, 20}$=0<br>p=0.995
	Time immobile	Periphery immobility time%	Center entries	Periphery time%	Center time%	
HI effect	$F_{1, 20}$=3.733<br>p=0.068	$F_{1, 20}$=3.657<br>p=0.07	$F_{1, 20}$=3.537<br>p=0.075	$F_{1, 20}$=0.696<br>p=0.414	$F_{1, 20}$=0.701<br>p=0.412	
Sex effect	$F_{1, 20}$=17.108<br>**p=0.001**	$F_{1, 20}$=16.967<br>**p=0.001**	$F_{1, 20}$=13.611<br>**p=0.001**	$F_{1, 20}$=8.709<br>**p=0.008**	$F_{1, 20}$=8.716<br>**p=0.008**	
HI*sex	$F_{1, 20}$=5.681<br>**p=0.027**	$F_{1, 20}$=5.569<br>**p=0.029**	$F_{1, 20}$=4.103<br>p=0.056	$F_{1, 20}$=2.763<br>p=0.112	$F_{1, 20}$=2.777<br>p=0.111	

$P < 0.05$ was considered statistically significant.

Bold values represent significant p-values.

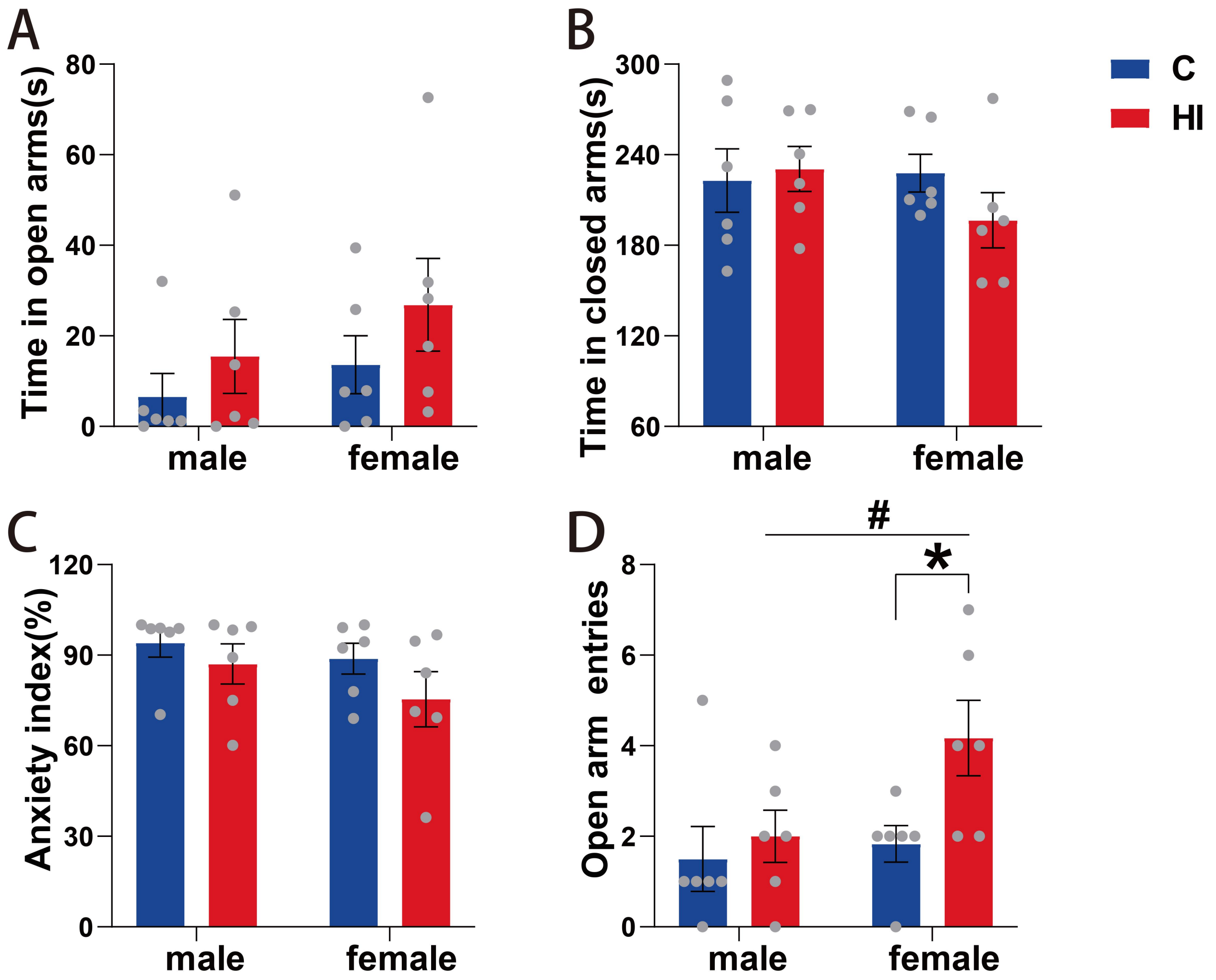

FIGURE S3

Evaluation of elevated plus-maze performance in PND63 rats. **(A)** Time in open arms. **(B)** Time in closed arms **(C)** Anxiety index **(D)** Number of open-arm entries. **(A-D)** Data are presented as means ± SEM (two-way ANOVA followed by the Bonferroni post hoc test). *$P < 0.05$; #$P < 0.05$. *: HI-female vs. C-female, HI-male vs. C-male; #: HI-female vs. HI-male (n = 6 per group).
